# Predicting ACI Outcomes with GMP In-Process Control Kinetic Metrics: Initial Chondrocyte Yield and Population Doubling Time as Prognostic Clinical Biomarkers

**DOI:** 10.3390/life16081297

**Published:** 2026-08-06

**Authors:** Virginie Philippe, Alexis E. Laurent, André Berchtold, Nathalie Hirt-Burri, Lee Ann Applegate, Robin Martin

**Affiliations:** 1 Orthopedics and Traumatology Service, Lausanne University Hospital, University of Lausanne, CH-1011 Lausanne, Switzerland; virginie.philippe@chuv.ch; 2 Manufacturing Department, LAM Biotechnologies SA, CH-1066 Epalinges, Switzerland; 3 Institute of Social Sciences, University of Lausanne, CH-1015 Lausanne, Switzerland; andre.berchtold@unil.ch; 4 Regenerative Therapy Unit, Plastic, Reconstructive and Hand Surgery Service, Lausanne University Hospital, University of Lausanne, CH-1066 Epalinges, Switzerland; nathalie.burri@chuv.ch (N.H.-B.); lee.laurent-applegate@unil.ch (L.A.A.); 5 Center for Applied Biotechnology and Molecular Medicine, University of Zurich, CH-8057 Zurich, Switzerland; 6 Oxford OSCAR Suzhou Center, Oxford University, Suzhou 215123, China; 7 Orthopedic Surgery Service, Fribourg Hospital, CH-1752 Villars-sur-Glâne, Switzerland; 8 Department of Surgical Specialties, Fribourg University, CH-1700 Fribourg, Switzerland

**Keywords:** autologous chondrocyte implantation, cartilage repair, knee, cell therapy, cryopreservation, Good Manufacturing Practices, human articular chondrocytes, patient-reported outcome measures, population doubling time, prognostic indicators

## Abstract

Purpose: To determine whether specific in vitro biologic characteristics and manufacturing kinetics of cultured human articular chondrocytes (HACs) can serve as predictive biomarkers for patient-reported and structural outcomes following second-generation autologous chondrocyte implantation (ACI) in the knee. Materials and Methods: This prospective cohort study evaluated 67 patients (mean age 25.1 ± 8.3 years) treated with second-generation ACI for large focal cartilage defects (mean size 5.4 ± 2.5 cm^2^) between 2017 and 2024. HACs were expanded under Good Manufacturing Practice (GMP) conditions using human platelet lysate-supplemented media. Four biological determinants were analyzed: (1) initial chondrocyte yield (ICY) isolated from the cartilage biopsy; (2) chondrogenic activity (relative *ACAN* and *COL2A1* expression); (3) final culture confluence level; and (4) HAC population doubling time (PDT) pre- and post-cryopreservation. Clinical outcomes (KOOS and IKDC) and MRI outcomes (MOCART) were assessed at 2 years (T1) and at a mean final follow-up of 4.2 ± 1.7 years (T2) via univariate and multiple regression analyses. Results: Static biosynthetic markers (*ACAN*/*COL2A1* expression) and final culture confluence did not significantly correlate with longitudinal clinical (KOOS/IKDC) or structural (MOCART) outcomes. Conversely, the retained kinetic parameters served as strong prognostic indicators. Multiple regression revealed that higher ICY values significantly predicted improvements across most KOOS subscales from baseline to T2, including KOOS Symptoms (β = 63.56; *p* < 0.01), KOOS Pain (β = 88.83; *p* < 0.001), KOOS ADL (β = 107.84; *p* < 0.001), and KOOS Sport and Recreation Function (β = 92.3; *p* < 0.05). Furthermore, a prolonged PDT, indicative of diminished in vitro proliferative vigor, inversely correlated with functional recovery in IKDC (β = −26.12; *p* < 0.01), KOOS Pain (β = −23.09; *p* < 0.05), and KOOS ADL (β = −21.71; *p* < 0.05) scores. Conclusions: The intrinsic proliferative vigor of the HAC cellular payload (shorter PDT) and higher initial cell yields are robust kinetic biomarkers associated with markedly superior mid-term clinical outcomes following second-generation ACI for large focal chondral defects in the knee. In contrast, standard morphological and gene expression metrics failed to predict in vivo functional success. These findings advocate for a risk-based paradigm shift in GMP quality control methodologies, emphasizing dynamic HAC growth kinetics over static cellular features to optimize patient-specific regenerative potential.

## 1. Introduction

Articular cartilage is a highly specialized, avascular, aneural, and alymphatic connective tissue [1,2]. Due to its isolation from systemic immune responses and blood-borne progenitor cells, focal chondral lesions possess a notoriously low intrinsic regenerative capacity [1,2]. Left untreated, localized defects alter joint kinematics, triggering a deleterious feed-forward loop of secondary synovial inflammation, extracellular matrix (ECM) degradation, and progressive joint degeneration [1,2].

To address symptomatic, full-thickness chondral defects, autologous chondrocyte implantation (ACI) has become a cornerstone of regenerative orthopedics [1,3,4]. ACI relies on the ex vivo amplification of a patient’s own dormant chondrocytes to biologically regenerate hyaline-like cartilage [4,5,6,7]. While longitudinal data demonstrates that second-generation ACI, which utilizes biocompatible scaffolds to foster a favorable microenvironment and ensure homogeneous spatial cell distribution, yields positive clinical outcomes, patient trajectories remain remarkably heterogeneous [2,3,6,8,9]. This variability strongly suggests that the mechanical advantages of the delivery vehicle are insufficient on their own; factors beyond surgical technique dictate graft success [5,6,10]. To date, prognostic research has heavily focused on surgical and epidemiological parameters, leaving the ‘black box’ of individual cellular behavior vastly underexplored [5,6].

A founding principle of advanced cell-based therapies is that treatment efficacy relies predominantly on the biological quality and resilience of the cellular payload [11,12,13]. During the mandatory ex vivo expansion within Good Manufacturing Practices (GMP)-regulated facilities, chondrocytes undergo significant physiological stress, including cryopreservation and monolayer-induced phenotypic drift (e.g., shifting from Type II to mechanically inferior Type I collagen production) [1,2,3,4,14,15,16]. Consequently, specific in vitro manufacturing parameters, such as the initial cell yield (ICY), population doubling time (PDT) pre- and post-cryopreservation, culture confluence, and the retention of chondrogenic markers, are heavily scrutinized [4,17,18,19]. Yet, the direct translational link between these isolated GMP metrics and long-term, in vivo patient-reported outcomes remains largely theoretical.

The objective of this study was to systematically investigate the complex relationship between several key biologic and kinetic characteristics of cultured human articular chondrocytes (HACs) and validated patient-reported clinical scores, alongside objective MRI-based structural outcomes, following second-generation ACI. Specifically, we meticulously analyzed a suite of critical process parameters (CPPs): the total number of chondrocytes isolated from the initial cartilage biopsy, their specific chondrogenic activity, the percentage of confluence at culture endpoint harvest, and the HAC batch PDT calculated both before and after cryopreservation. Our goal was to determine if these specific in vitro manufacturing parameters can be reliably established as prognostic indicators of clinical success and cartilage durability in the short- and mid-term.

This study’s primary novelty and specific clinical significance lie in its ability to draw a direct, empirical correlation between highly controlled, in vitro GMP cell manufacturing parameters and long-term clinical realities within a robust prospective cohort of ACI patients. While the bulk of previous research has concentrated either purely on the technical, bench-top aspects of cell expansion or on isolated short-term clinical results, this work bridges the translational divide [4]. It provides a direct, data-driven link between specific GMP-controlled cell metrics, specifically ICY and proliferative capacity both pre- and post-cryopreservation, and patient-reported trajectories of pain, joint function, and quality of life up to five years post-surgery. This supports the overarching hypothesis that both intrinsic biological traits and extrinsic factors related to GMP cell manufacture dictate the ultimate biologic quality of the therapeutic cells. By identifying these crucial factors for ACI success, this study provides a robust scientific basis for the stringent selection and refinement of key and critical manufacturing process parameters. Furthermore, it underscores that the strict standardization of HAC culture and cryopreservation conditions is primordial to optimize PDT values, thereby unlocking the potential for a far more predictable, efficacious, and highly personalized approach to ACI [4,20,21].

## 2. Materials and Methods

### 2.1. Study Design and Patient Population

This research was conducted as a prospective clinical cohort study evaluating patients who underwent second-generation ACI for the treatment of focal articular cartilage defects of the knee between 2017 and 2024 [4]. The clinical trial protocol was rigorously reviewed and approved by the competent regulatory and ethical authorities, specifically Swissethics (CER-VD #2015-00145) and Swissmedic (Registration #2016TpP1005), and was formally registered on ClinicalTrials.gov (Identifier: NCT04296487). Prior to any study-specific interventions, comprehensive written informed consent was obtained from all participating individuals.

The study population consisted predominantly of young patients because ACI is indicated for the treatment of focal, well-demarcated cartilage defects and is not intended for patients with degenerative osteoarthritis. Strict eligibility criteria were established to ensure cohort homogeneity and isolate the relevant biological variables. Inclusion criteria mandated that participants be skeletally mature individuals presenting with symptomatic, full-thickness focal chondral or osteochondral lesions of the knee joint. Pathological lesions were required to possess a surface area of ≥2 cm^2^, and patients were required to have a body mass index (BMI) of <35 kg/m^2^.

**Figure 1:**
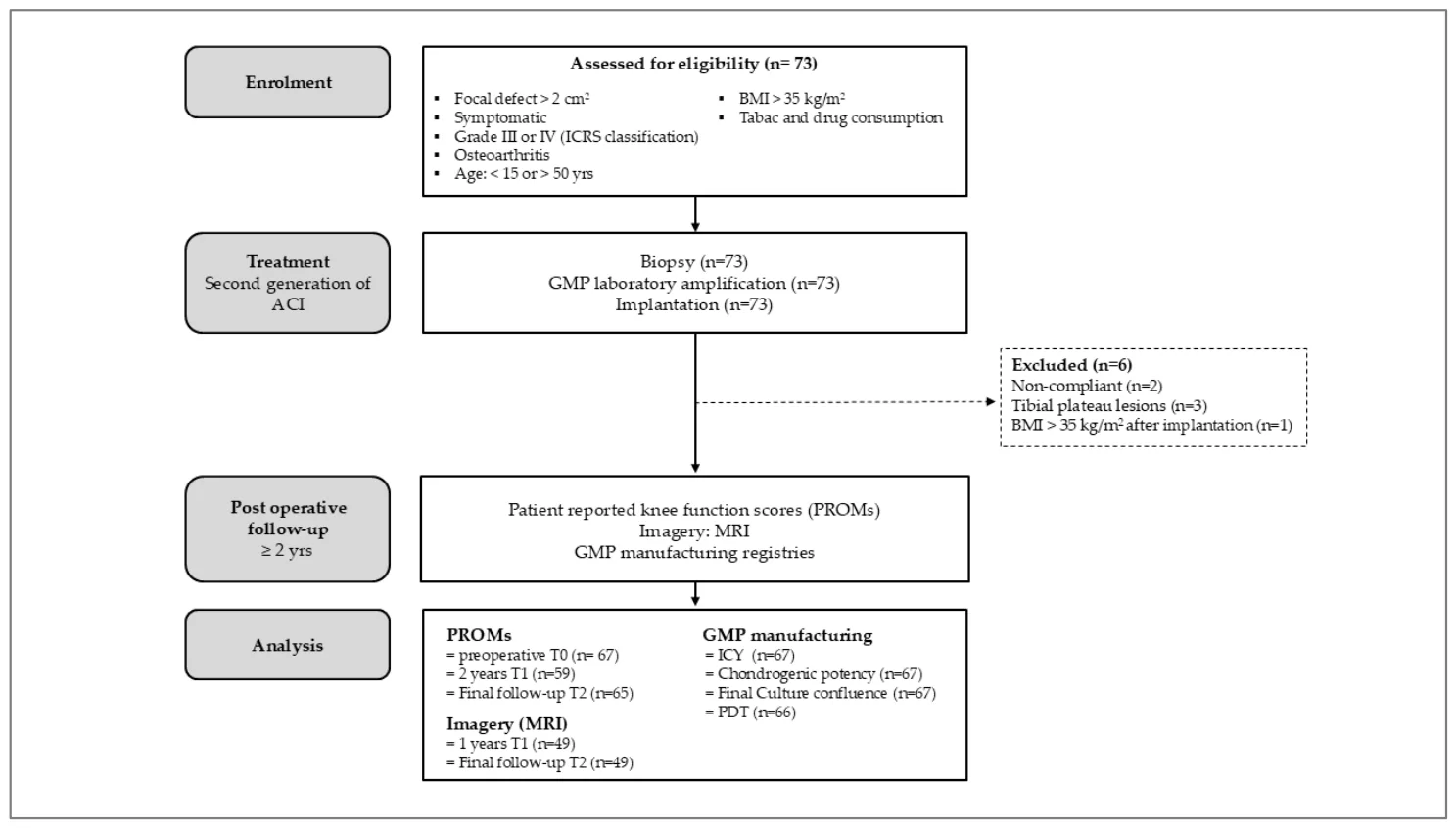
Study flowchart and patient disposition. Of the 73 patients initially assessed and treated with second-generation autologous chondrocyte implantation (ACI), a total of 6 were excluded due to protocol non-compliance (n = 2), tibial plateau lesions (n = 3), or a postoperative BMI exceeding 35 kg/m^2^ (n = 1). The final analytical cohort comprised 67 patients evaluated postoperatively for at least 2 years. Clinical efficacy was quantified using patient-reported outcome measures (PROMs) at baseline (T0, n = 67), 2 years (T1, n = 59), and at final follow-up (T2, n = 65). Structural integration was assessed via MRI at 1 year (T1, n = 49) and at final follow-up (T2, n = 49). GMP manufacturing parameters, including the initial chondrocyte yield (ICY), chondrogenic potency, and final culture confluence, were analyzed for the entire retained cohort (n = 67), whereas the population doubling time (PDT) was analyzed for 66 patients. Formal regression modelling was performed on patients with complete datasets, including 54 patients for the IKDC, 45 patients for the MOCART, and 53 patients for all other analyses.

Patients were subsequently excluded from the study cohort if they met any of the following criteria: chronological age outside the defined therapeutic window of 15 to 50 years, radiological or clinical evidence of generalized osteoarthritis, a history of active smoking, or documented illicit substance abuse. Furthermore, patients who failed to complete a minimum postoperative clinical follow-up of 2 years were excluded from the final statistical analysis. Following the rigorous application of these criteria, the definitive study cohort comprised 67 patients (38 males, 29 females), demonstrating a mean longitudinal follow-up duration of 4.2 ± 1.7 years (Figure 1).

### 2.2. Surgical Procedure and GMP Cell Therapy Manufacturing Process

The strictly controlled GMP bioprocessing protocol for the studied cellular therapy has been detailed extensively in previous reports and is summarized herein [4]. The clinical intervention necessitated a meticulously staged, two-part surgical approach. The initial phase consisted of an arthroscopic harvest of pristine articular cartilage, yielding a standard biopsy measuring approximately 4 × 12 mm, from the most proximal part of the medial trochlea groove (n = 58), intercondylar notch (n = 8), or both (n = 1). Concurrently, peripheral blood was drawn to facilitate the derivation of autologous serum. Both the cartilage tissue and blood specimens were immediately secured in sterile, temperature-validated transport receptacles and transferred under strict logistical control to our centralized, in-house GMP manufacturing facility.

Upon transfer to the cleanroom environment, the cartilage biopsy was subjected to a standardized, two-stage enzymatic digestion protocol to liberate HACs from the dense ECM. The macroscopic tissue was initially minced manually with a sterile scalpel into micro-fragments ≤ 1 mm^3^. These fragments were sequentially digested using a pronase solution (Sigma-Aldrich, Buchs, Switzerland) for 1 h at 37 °C, followed by an overnight incubation in a type II collagenase solution (Thermo Fisher Scientific, Waltham, MA, USA), maintained continuously at 37 °C under gentle agitation. The ensuing HAC cell suspension was subsequently filtered to remove undigested matrix debris, washed via centrifugation, and subjected to rigorous quantification and viability assays. An initial viability threshold exceeding 90% served as a critical in-process control (IPC) and a mandatory acceptance criterion prior to in vitro culture initiation.

The successfully isolated HACs, constituting the primary cellular starting material, were seeded for monolayer expansion under fully GMP-compliant atmospheric conditions. The basal culture medium (DMEM–Ham’s F12 [1:1, Sigma-Aldrich, Buchs, Switzerland] with ascorbic acid 25 µg/mL [Streuli Pharma, Uznach, Switzerland]) was supplemented with 10% pooled allogeneic hPL (Sexton Biotechnologies, Indianapolis, IN, USA), which served as a highly validated, scalable, and standardized alternative to autologous serum supplementation [4]. Upon reaching target confluence in 10–15 days, the primary HAC monolayers were enzymatically detached, quantified, and cryopreserved in validated aliquots at a standardized cellular density of (1.0 ± 0.2) × 10^6^ cells/mL. This cryopreserved intermediate constituted the patient-specific Master Cell Bank (MCB).

Notably, during the clinical timeline of this cohort, a forced transition in the GMP-certified cryopreservation medium occurred. Due to the commercial discontinuation of the legacy reagent (Biofreeze, Sigma-Aldrich, Buchs, Switzerland), the protocol was prospectively adapted to utilize a supplier-validated alternative (CryoSOfree, Sigma-Aldrich, Buchs, Switzerland). To generate the definitive therapeutic cell lot, an aliquot from the MCB was thawed and subjected to a secondary, final monolayer expansion phase. Upon reaching final harvest criteria in 6–7 days, the expanded HACs were formulated into an injectable suspension at a defined concentration of 3 × 10^4^ cells/µL in 0.9% NaCl (Bichsel, Unterseen, Switzerland) with 20% autologous serum, guaranteeing a consistent, standardized volumetric dose for the patient.

The second surgical stage, the definitive implantation, was executed via an open arthrotomy, typically occurring approximately 3.5 months post-biopsy. The focal chondral defect was meticulously debrided to establish a geometrically stable, vertical cartilaginous perimeter and a healthy subchondral bed. A biocompatible collagen membrane (Chondro-Gide^®^, Geistlich Pharma, Wolhusen, Switzerland) was then precisely tailored and sutured or glued to create a watertight compartment. Finally, the autologous HAC suspension was injected directly into the sealed defect site at a targeted therapeutic dose of 2 × 10^6^ cells/cm^2^. The details about the rehabilitation program subsequently followed by the study participants are presented in Table S3 and were based on a critical review of the literature [22,23,24].

Throughout the biomanufacturing pipeline, the PDT was rigorously calculated for each patient-specific HAC cell type, both pre- and post-cryopreservation, to quantitatively assess cellular kinetic vigor and define optimal biological quality using the following equation:

$$PDT=\frac{T\cdot ln\left(2\right)}{ln(\frac{Nf}{N0})}$$

where T was the culture duration in days, Nf the final number of harvested cells, and N0 the initial number of seeded cells. The continuous, longitudinal monitoring of this specific kinetic parameter proved indispensable for evaluating the physiological impact of the aforementioned extrinsic transition in cryoprotectant media mandated during the study period.

### 2.3. Biologic Determinants and Outcome Measures

To identify potential in vitro correlates of clinical efficacy, four fundamental biological parameters, serving as surrogates for cellular potency and metabolic vigor, were extracted from the available GMP manufacturing registries:

(1) Initial Chondrocyte Yield (ICY): Defined as the absolute count of viable human articular chondrocytes (HACs) recovered immediately following the standardized enzymatic digestion of the primary cartilage biopsy, prior to any ex vivo expansion.
(2) Chondrogenic Potency Assays: HAC functional redifferentiation potential was evaluated using a 3D cell pellet culture model. Transcriptional profiling of hallmark chondrogenic markers, specifically aggrecan (*ACAN*; primer sequence F 5′ GGT ACC AGT GCA CAG AGG GGT T 3′; R 5′ TGC AGG TGA TCT GAG GCT CCT 3′) and collagen Type II, Alpha 1 chain (*COL2A1*; primer sequence F 5′ GGC AAT AGC AGG TTC ACG TAC 3′; R 5′ GAT AAC AGT CTT GCC CCA CTT 3′), was quantified via real-time quantitative polymerase chain reaction (RT-qPCR) as described elsewhere [4]. Briefly, RNA quantity and purity were assessed using spectrophotometric measurements (NanoDrop, Thermo Fisher Scientific, Waltham, MA, USA). Samples were considered suitable for downstream RT-qPCR analysis when A_260_/A_280_ ratios were within 1.8–2.1 and A_260_/A_230_ ratios were ≥1.8, indicating acceptable RNA purity. Fluorescence was acquired using the following cycling conditions: 95 °C for 3 min (enzyme activation) and 40 amplification cycles (95 °C for 3 s and annealing extension at 60 °C for 30 s). Each sample was run in triplicate, and the relative expression level for each gene was normalized to *GAPDH* (primer sequence F 5′ CGC TCT CTG CTC CTC CTG TT 3′; R 5′ CCA TGG TGT CTG AGC GAT GT 3′). Relative gene expression was calculated using the 2^−ΔΔCt^ method. For the reverse transcription, PrimeScript RT reagent kits were procured from Takara Bio, San Jose, CA, USA. The KAPA SYBR Fast qPCR kit was procured from Kapa Biosystems, Roche, Basel, Switzerland. Primers were procured from Microsynth, Balgach, Switzerland. The QuantStudio™ 3 Real-time PCR system (Thermo Fisher Scientific, Waltham, MA, USA) was used for the study.
(3) Terminal Culture Confluency: A semi-quantitative assessment of the cellular surface coverage within the expansion vessels was made at the time of harvest, serving as a surrogate metric for spatial growth kinetics and density-dependent phenotypic stability. The cell confluency was recorded in the form of photomicrographs and was determined visually by two experienced GMP cell manufacturing operators during terminal culture vessel examination.
(4) Population Doubling Time (PDT): A dynamic measure of proliferative vigor, it was calculated as the mean time required for the cell population to double in number during the in vitro expansion phase. The PDT was rigorously quantified both pre- and post-cryopreservation to assess the impact of the aforementioned transition in cryoprotectant agents on cellular recovery and kinetic momentum.

These biological CQAs were selected to provide a multi-dimensional profile of the cellular payload, encompassing absolute biomass, proliferative vigor, and biosynthetic fidelity.

Clinical success was evaluated through a bimodal framework of subjective and objective metrics. PROMs included the KOOS and the IKDC Subjective Knee Evaluation Form. The KOOS was analyzed across its five validated subscales: Pain (KOOS P), other Symptoms (KOOS S), Activities of Daily Living (KOOS ADL), Sport and Recreation Function (KOOS Sport), and knee-related Quality of Life (KOOS QOL).

Radiological outcomes were objectively quantified using the MOCART score evaluated by a single blinded osteo-articular radiologist, which then provided a high-resolution morphological assessment of graft integration, surface congruity, and subchondral integrity. Longitudinal evaluations were conducted at two distinct postoperative intervals: an initial short-term follow-up at 2 years (T1) and a mid-to-long-term follow-up around 4 years (T2). The influence of manufacturing-level variables, specifically the alteration in cryopreservation media, was systematically analyzed relative to these clinical and structural trajectories.

### 2.4. Statistical Analyses

In addition to standard descriptive analyses, the analytical framework of this study employed both univariate and multiple linear regression models to systematically identify the potential correlations between the four primary biological determinants (ICY, chondrogenic activity, final culture confluence, and PDT) and the longitudinal clinical and structural outcomes (KOOS subscales, IKDC, and MOCART). To account for inter-patient differences at baseline, we systematically considered the changes observed between baseline and T1 on the one hand and between baseline and T2 on the other. While the total prospective cohort comprised 67 subjects, the formal regression modelling was generally restricted to a subset of 53 patients (i.e., n = 54 for IKDC, n = 45 for MOCART) to maintain the integrity of the multidimensional dataset by excluding cases with missing data for specific variables. The optimal PDT threshold was identified using an empirical approach: graphs were plotted showing the relationship between the five KOOS measures and PDT, and the probability of a favorable outcome was calculated by varying the threshold value until the optimal value was found. A similar approach was applied in the case of the ICY. The type-I error was set at 0.05 for all computations. The open-source statistical environment R was used for all computations (R Core Team, R Foundation for Statistical Computing, Vienna, Austria, 2025).

## 3. Results

### 3.1. Cohort Characteristics and Patient-Reported Outcomes

A total of 67 patients were included in the study, with a mean follow-up duration of 4.2 ± 1.7 years, with a subset of 3.6 ± 1.2 years for MRI follow-up. The baseline demographic and clinical characteristics of the cohort are summarized in Table 1.

The study population primarily consisted of a young demographic, representative of the typical etiology for these articular pathologies, presenting with focal joint lesions that encompassed both isolated chondral and osteochondral defects (Table 1). Importantly, the mean defect size in the present cohort was larger (>4 cm^2^) than in most clinical reports on ACI. Furthermore, osteotomies were systematically performed when clinically indicated for compartment unloading, prior to ACI interventions.

Clinical efficacy was evaluated using highly validated Patient-Reported Outcome Measures (PROMs), specifically the Knee Injury and Osteoarthritis Outcome Score (KOOS) and the International Knee Documentation Committee (IKDC) Subjective Knee Evaluation Form. These instruments provide a robust, quantitative assessment of the patient’s joint-specific pain, functional capacity, and overall quality of life. The KOOS profile captured five distinct subscales: Pain, other Symptoms, Activities of Daily Living (ADL), Sport and Recreation Function (Sport), and knee-related Quality of Life (QOL). Concurrently, the IKDC score provided a comprehensive evaluation encompassing symptoms, joint function, and sports activity level.

**Figure 2:**
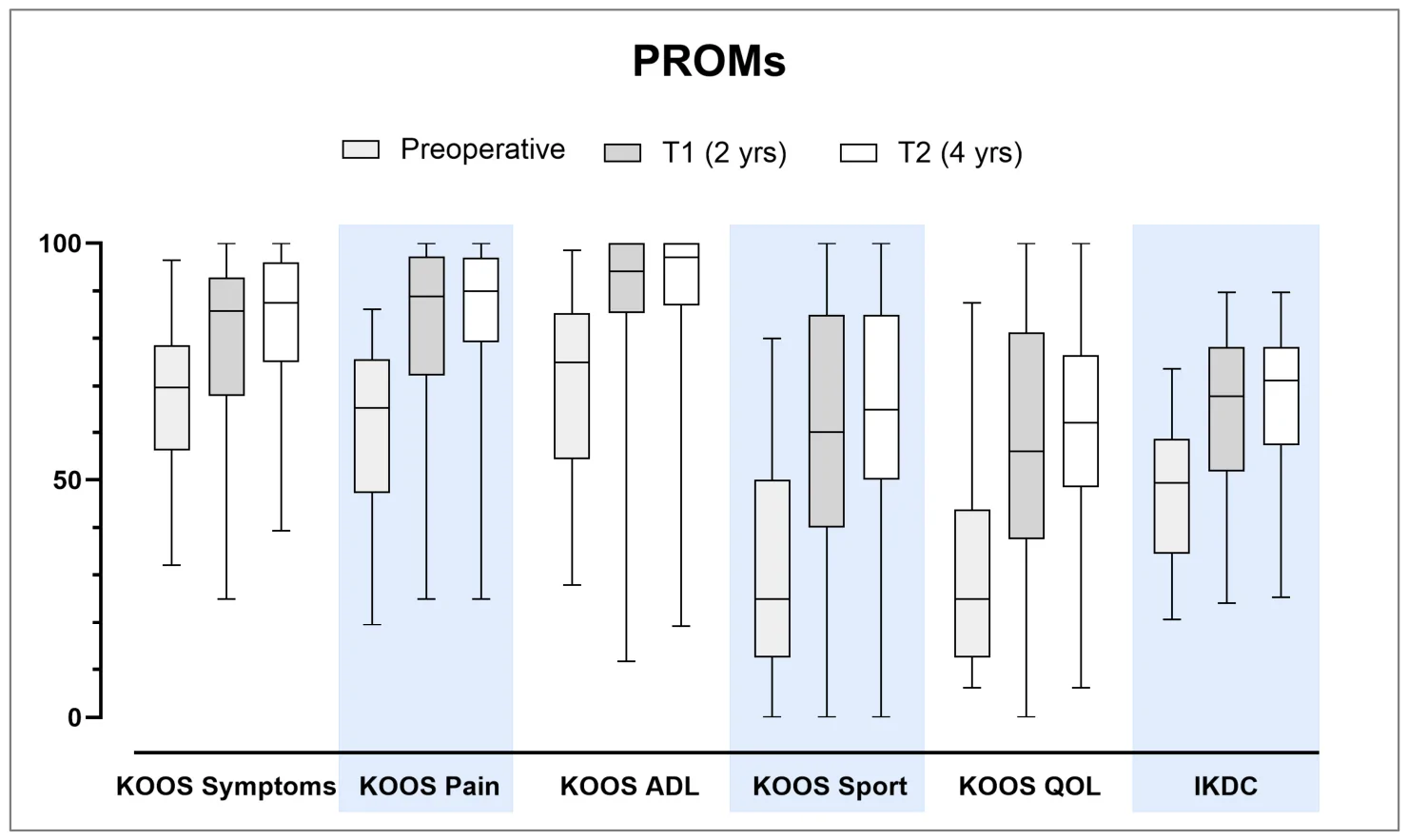
Longitudinal progression of patient-reported outcome measures (PROMs) following second-generation autologous chondrocyte implantation. Box-and-whisker plots illustrate the temporal evolution of the five Knee Injury and Osteoarthritis Outcome Score (KOOS) subscales (Symptoms, Pain, Activities of Daily Living [ADL], Sport, Quality of Life [QOL]) and the International Knee Documentation Committee (IKDC) subjective scores. Clinical assessments were performed preoperatively (baseline, n = 67), at the intermediate 2-year follow-up (T1, n = 59), and at the final ~4-year follow-up (T2, n = 65). Within each box, the central horizontal line represents the median, the upper and lower box boundaries indicate the interquartile range (IQR), and the whiskers denote the data range (minimum and maximum values).

Assessments were conducted pre-operatively (baseline) and were then prospectively recorded at 2- and 4-year follow-up intervals following ACI. Analysis of these clinical endpoints revealed a highly favorable postoperative trajectory across the cohort. Patients demonstrated substantial clinical improvements across all KOOS subscales and IKDC scores at the initial 2-year follow-up (T1). Furthermore, this positive clinical evolution was not only sustained but exhibited continued, progressive improvement across the majority of the functional endpoints at the 4-year follow-up mark (T2, Figure 2).

In addition to the overall KOOS and IKDC scores presented in Figure 2, magnetic resonance observation of cartilage repair tissue (MOCART) data gathered for the cohort is presented in the Supplementary Materials (Figure S1). Of the 67 patients initially enrolled in the study, 14 were excluded from the multiple regression analysis due to missing data for specific biological or clinical variables. To ensure no selection bias, a comparative analysis was performed between the regression subset (n = 53) and the excluded subset (n = 14). No statistically significant differences were observed in mean age (24.8 vs. 26.2 years), mean defect size (5.3 vs. 5.7 cm^2^), or baseline KOOS/IKDC scores (*p* > 0.05 for all), confirming that the regression cohort was representative of the total study population. Overall, it was confirmed that the demographic parameters of the cohort did not act as confounding factors for PROMS and MOCART data for the studied cohort.

### 3.2. Biological Endpoints Analyzed from Patient Chondral Biopsies and Chondrocyte Culture

**Figure 3:**
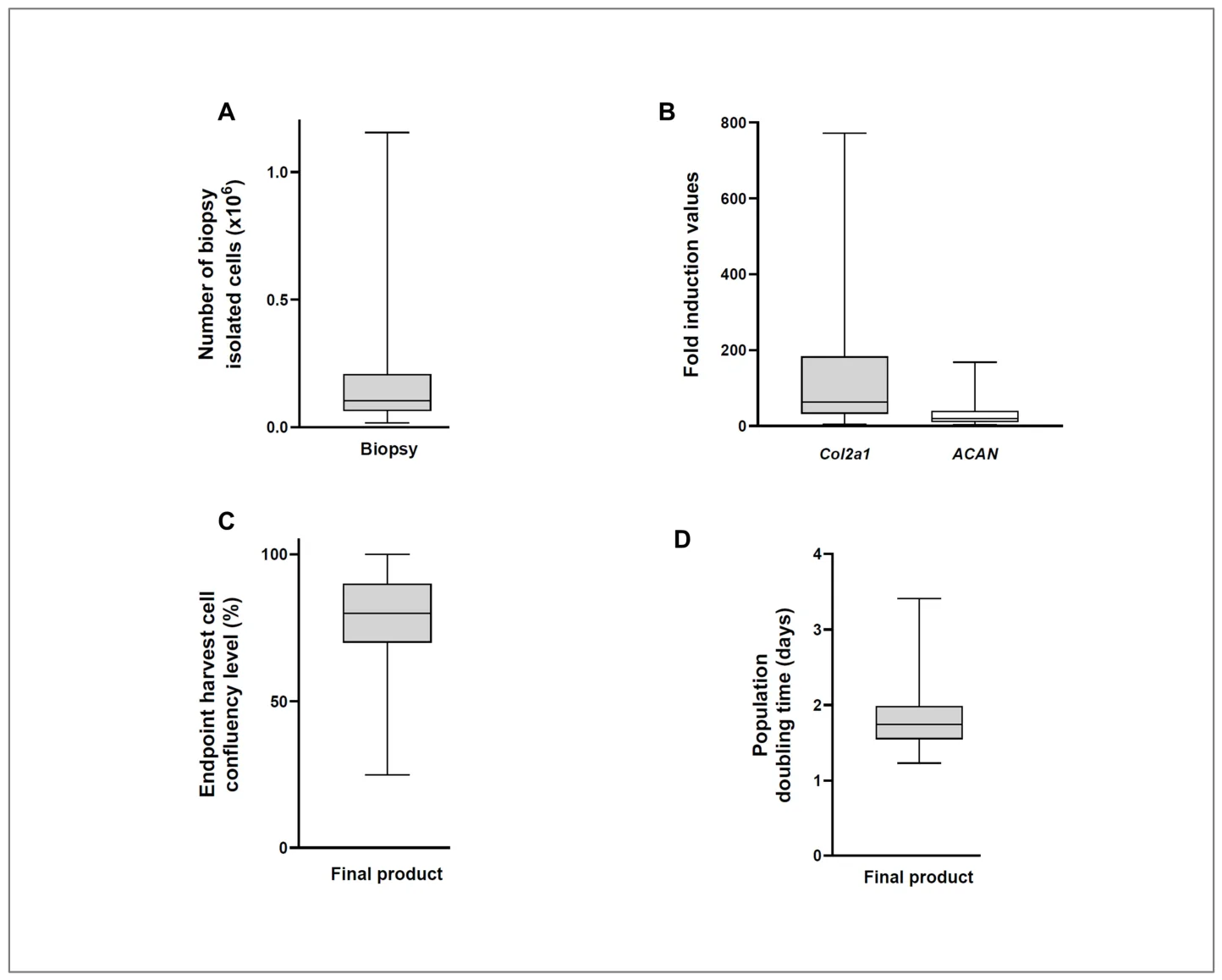
Characterization of cellular kinetics and biological quality from the initial cartilage biopsy to the final cell therapy product (n = 67). Box-and-whisker plots depicting key manufacturing parameters of human articular chondrocytes (HACs) during ex vivo GMP expansion. (**A**) Absolute number of cells isolated following the initial cartilage biopsy digestion (×10^6^) (n = 67). (**B**) Chondrogenic potency, assessed via the relative fold induction (normalized to the *GAPDH* housekeeping gene) of *COL2A1* and *ACAN* gene expression in HACs cultured in a 3D cell pellet quality control model (n = 67). (**C**) Percentage of cellular confluence at the endpoint HAC harvest (n = 67). (**D**) Population doubling time values (PDT, expressed in days) of the final HAC cellular suspension prior to implantation (n = 66). Within the box plots, central horizontal lines represent the median, horizontal box boundaries indicate the interquartile range (IQR), and whiskers demonstrate the full data range (minimum to maximum). *COL2A1*, gene coding type II collagen alpha 1 chain; *ACAN*, gene coding aggrecan.

Several critical biological parameters were quantified during the ex vivo GMP expansion of patient-derived chondrocytes to assess cell quality and viability. These metrics included the ICY from the cartilage biopsies, HAC culture confluency at the time of harvest, PDT values both pre- and post-cryopreservation, and the retention of chondrogenic potential. Following the enzymatic digestion of the initial chondral biopsies, the absolute cell yield demonstrated substantial inter-patient variability (Figure 3A).

To evaluate the functional phenotype and matrix-producing capability of the cells, the cryopreserved and expanded chondrocytes were transitioned into a 3D cell culture system. The fold induction of key chondrogenic ECM genes, specifically *COL2A1* and *ACAN*, was subsequently quantified via RT-qPCR to confirm chondrogenic potential (Figure 3B). Cellular growth kinetics were further characterized by assessing the endpoint confluency of the HAC cultures at the time of final harvest before therapeutic use (Figure 3C). The final PDT values of the active substance used in the cell-based product which was implanted in the patients are illustrated in Figure 3D.

A secondary analysis was conducted to determine if the initial cellular biomass size influenced subsequent expansion kinetics. No significant inverse correlation was observed between the ICY value and the PDT of the final cell therapy product (r = −0.11, *p* = 0.399, n = 66). This suggests that cartilage biopsies yielding a lower number of initially isolated cells do not automatically possess a reduced proliferative vigor (due to baseline cellular stress or a localized pathological ‘field effect’ within the donor joint).

### 3.3. Lack of Association Between Classical Chondrogenic Markers and Clinical Outcomes

Statistical analysis revealed no significant correlations between any of the four primary biologic determinants and either the IKDC or MOCART scores at the 2-year (T1) or 4-year (T2) follow-up intervals. Furthermore, univariate analysis evaluating the relationship between these in vitro biological variables and the change in clinical scores from baseline (T0) to T2, as well as absolute MOCART scores at T2, demonstrated no significant predictive value for *ACAN* or *COL2A1* relative gene expression.

Similarly, the final HAC culture confluence percentage lacked statistically significant associations with the majority of the KOOS subscales, despite some observable, albeit non-significant, trends. Collectively, these findings indicate that in vitro chondrogenic marker expression and culture confluence metrics were not reliable prognostic indicators of long-term clinical or structural outcomes within this specific cohort.

### 3.4. Association Between Initial Chondrocyte Yield and Clinical Outcomes

During the early follow-up interval (T0–T1), none of the four investigated biological parameters exhibited a statistically significant association with changes in clinical scores. Conversely, multiple regression analysis demonstrated that a higher ICY value (i.e., >10^5^ isolated cells, threshold determined by statistical analysis) was significantly associated with longitudinal improvements across the majority of KOOS subscales at the extended 4-year follow-up (T0–T2), with the exception of the KOOS Quality of Life (QOL) subscale (Table S1). Specifically, significant positive associations were observed for KOOS Symptoms (β = 63.56; *p* < 0.01), KOOS Pain (β = 88.83; *p* < 0.001), KOOS ADL (β = 107.84; *p* < 0.001), and KOOS Sport (β = 92.30; *p* < 0.05, Table S1).

**Figure 4:**
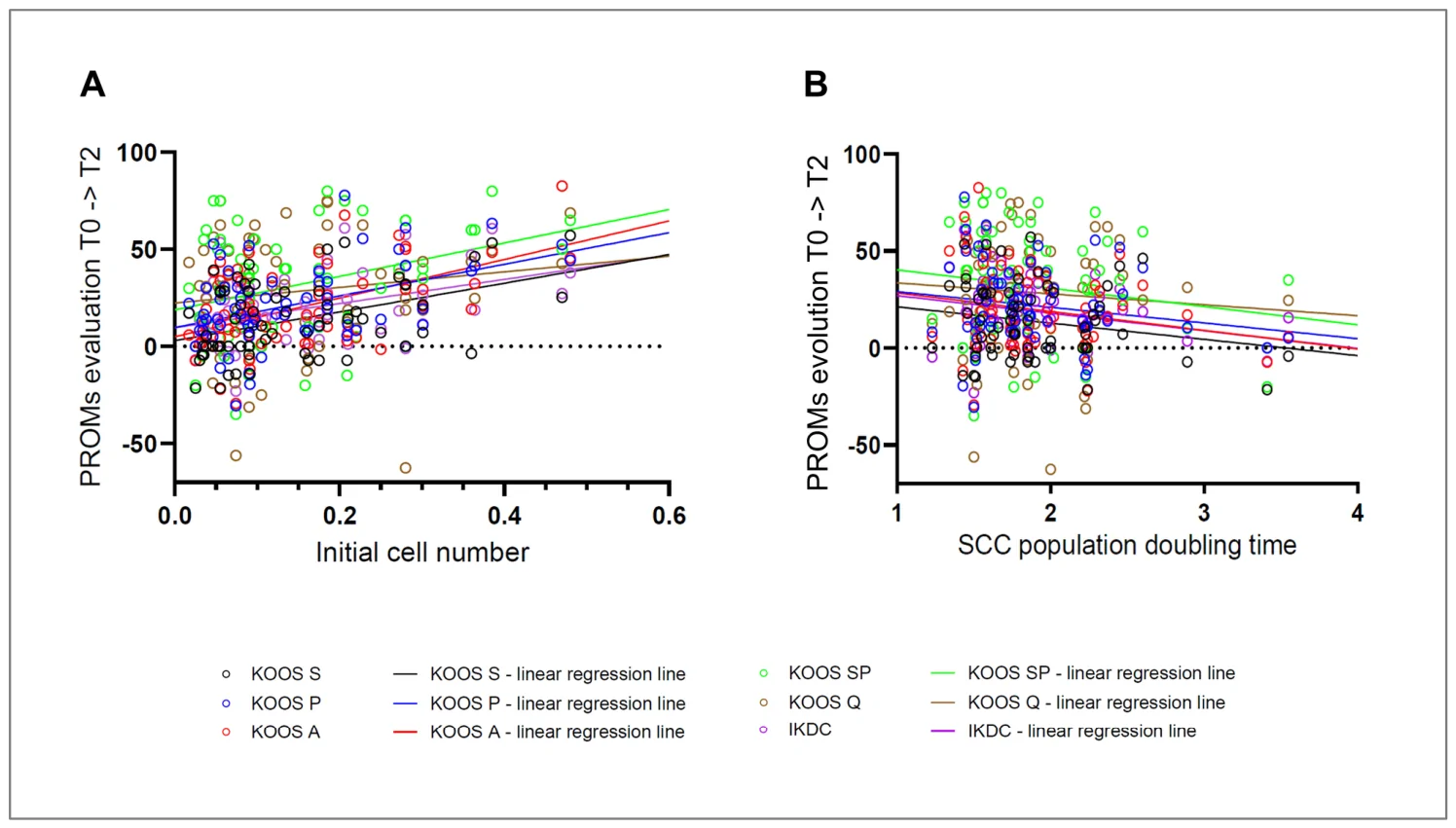
Correlation between ex vivo cellular kinetic parameters and longitudinal changes in patient-reported clinical outcomes. Scatter plots illustrating the relationship between (**A**) the initial chondrocyte yield (ICY) obtained from the surgical cartilage biopsy and (**B**) the population doubling time (PDT, in days) of the final chondrocyte cell suspension (SCC) prior to implantation, plotted against the absolute change in clinical scores (ΔPROMs) from baseline (T0) to the final 4-year follow-up (T2). Individual patient data points are displayed alongside corresponding simple linear regression lines for each clinical subscale (color correspondence). KOOS, Knee Injury and Osteoarthritis Outcome Score (S, Symptoms; P, Pain; ADL, Activities of Daily Living; SP, Sport and Recreation Function; Q, Quality of Life); IKDC, International Knee Documentation Committee score; SCC, chondrocyte cell suspension.

To further contextualize the translational impact of ex vivo GMP cell manufacturing metrics on longitudinal clinical efficacy, patient-specific changes in functional outcomes were visually plotted against key biological parameters. Figure 4 delineates the absolute changes in comprehensive PROMs (ΔPROMs), encompassing all five KOOS subscales and the IKDC score from baseline (T0) to the 4-year final follow-up (T2), relative to the two identified critical cellular characteristics (ICY, PDT).

Graphical representation of the data, where individual patient change scores are plotted alongside simple linear regression lines, corroborates this positive association between ICY and PROMs improvement, albeit with expected inter-patient variability (Figure 4). Specifically, these scatter plots illustrate the clinical trajectories correlated with the ICY obtained from the surgical cartilage biopsy (Figure 4A) alongside the proliferative kinetics, quantified as the PDT, of the definitive chondrocyte cell suspension (SCC) immediately prior to surgical implantation (Figure 4B). These findings indicate that the functional impact of the initial biopsy cell yield on postoperative pain relief and joint restoration following ACI becomes progressively more pronounced over a longer clinical trajectory (Figure 4).

**Figure 5:**
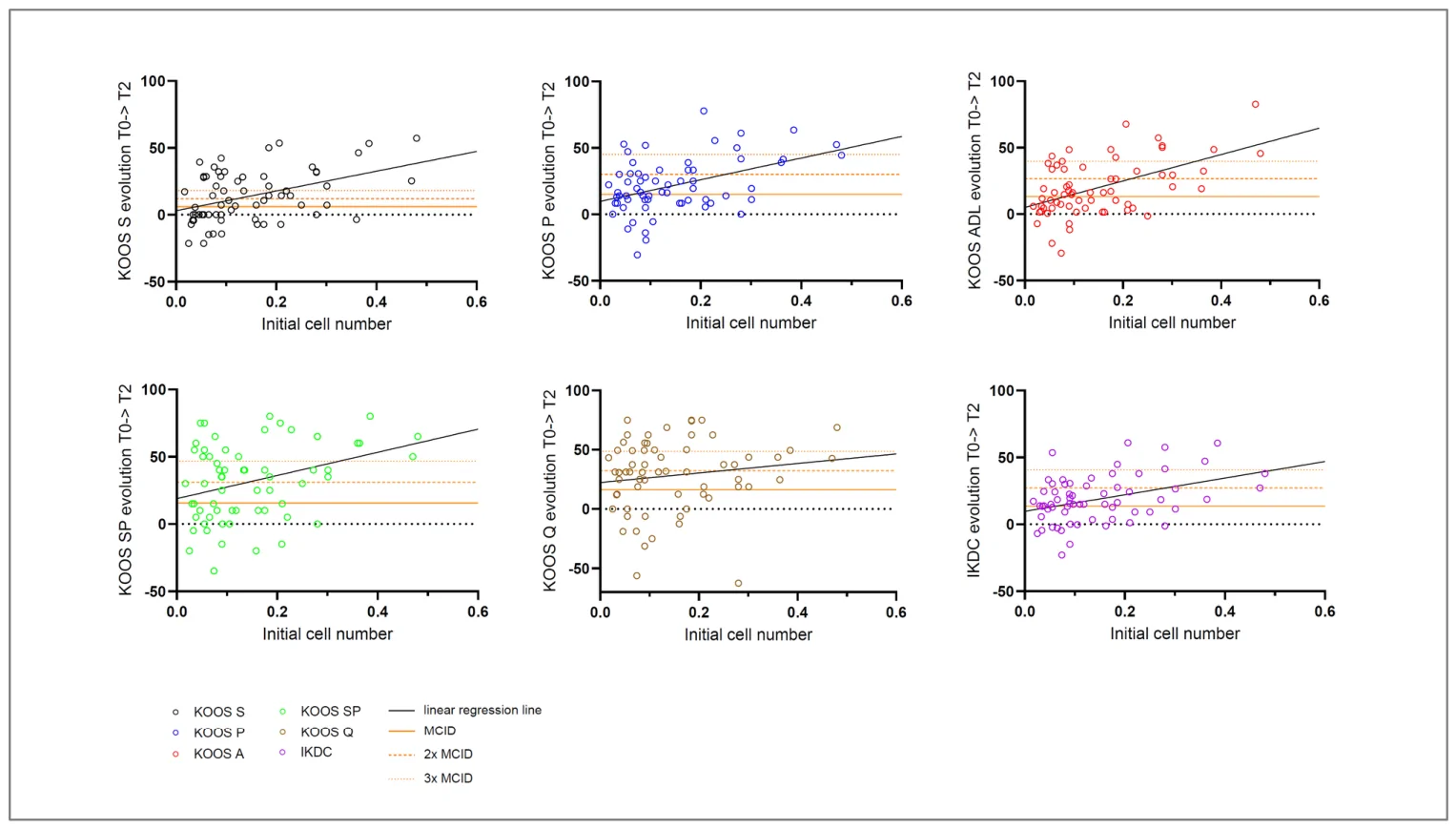
Influence of initial chondrocyte yield (ICY) on achieving clinically meaningful functional improvements. Scatter plots detailing the relationship between the absolute number of chondrocytes isolated from the initial cartilage biopsy (ICY) and the longitudinal change in clinical scores (ΔPROMs) from baseline (T0) to the final 4-year follow-up (T2). Each panel represents a distinct functional metric (five KOOS subscales and the IKDC score). Individual patient trajectories are plotted alongside simple linear regression lines. The black dotted horizontal line denotes the preoperative baseline (ΔPROM = 0). Solid and dashed orange lines represent the established Minimal Clinically Important Difference (MCID) and its multiples (2× and 3× MCID), delineating objective thresholds for substantial clinical benefits. KOOS, Knee Injury and Osteoarthritis Outcome Score (S, Symptoms; P, Pain; ADL, Activities of Daily Living; SP, Sport and Recreation Function; Q, Quality of Life); IKDC, International Knee Documentation Committee; MCID, Minimal Clinically Important Difference.

To provide a more granular visualization of the relationship between the ICY and long-term functional recovery, patient-specific clinical trajectories were mapped directly against the absolute starting chondrocyte count. An analysis of the data distribution relative to baseline (ΔPROM = 0) and minimal clinically important difference (MCID) thresholds suggests that an ICY value exceeding 10^5^ cells may increase the probability of achieving a clinically meaningful outcome. Importantly, although the existence of this 10^5^ threshold has not been proven theoretically, it has been confirmed experimentally based on our data. Figure 5 details the magnitude of change (ΔPROM) across all five KOOS subscales and the overall IKDC score from baseline (T0) to the final 4-year follow-up (T2).

Crucially, the incorporation of established MCID thresholds, alongside multiples thereof (2× and 3× MCID), allows for a highly nuanced assessment of clinical efficacy. This visual representation not only reinforces the overarching positive correlation between higher ICY values and improved patient-reported outcomes, but also clearly delineates the proportion of the cohort achieving distinct, clinically meaningful milestones in their postoperative recovery (Figure 5).

### 3.5. Association Between HAC Cell Population Doubling Time and Clinical Outcomes

Consistent with the early follow-up findings for ICY, no statistically significant relationships were observed between the PDT and changes in clinical scores during the initial postoperative interval (T0–T1). However, longitudinal analysis revealed that the PDT exerted a significant influence on clinical trajectories over the extended follow-up period (T0–T2).

Across the entire cohort, a prolonged PDT, indicative of diminished cellular proliferative capacity during the ex vivo GMP expansion phase, was inversely correlated with functional improvements from baseline to the final 4-year follow-up (T0–T2). Specifically, slower proliferation rates were significantly associated with an attenuated clinical evolution in the overall IKDC score (β = −26.12; *p* < 0.01), KOOS Pain (β = −23.09; *p* < 0.05), and KOOS ADL (β = −21.71; *p* < 0.05, Table S1).

Collectively, these findings indicate that the inherent proliferative potential of HACs is a critical determinant of functional recovery that becomes progressively more apparent over an extended clinical timeline. Consequently, sluggish in vitro expansion kinetics serve as a prognostic indicator for inferior long-term clinical outcomes following ACI (Figure 4).

### 3.6. PDT Threshold and Probability of Clinically Meaningful Improvement

**Figure 6:**
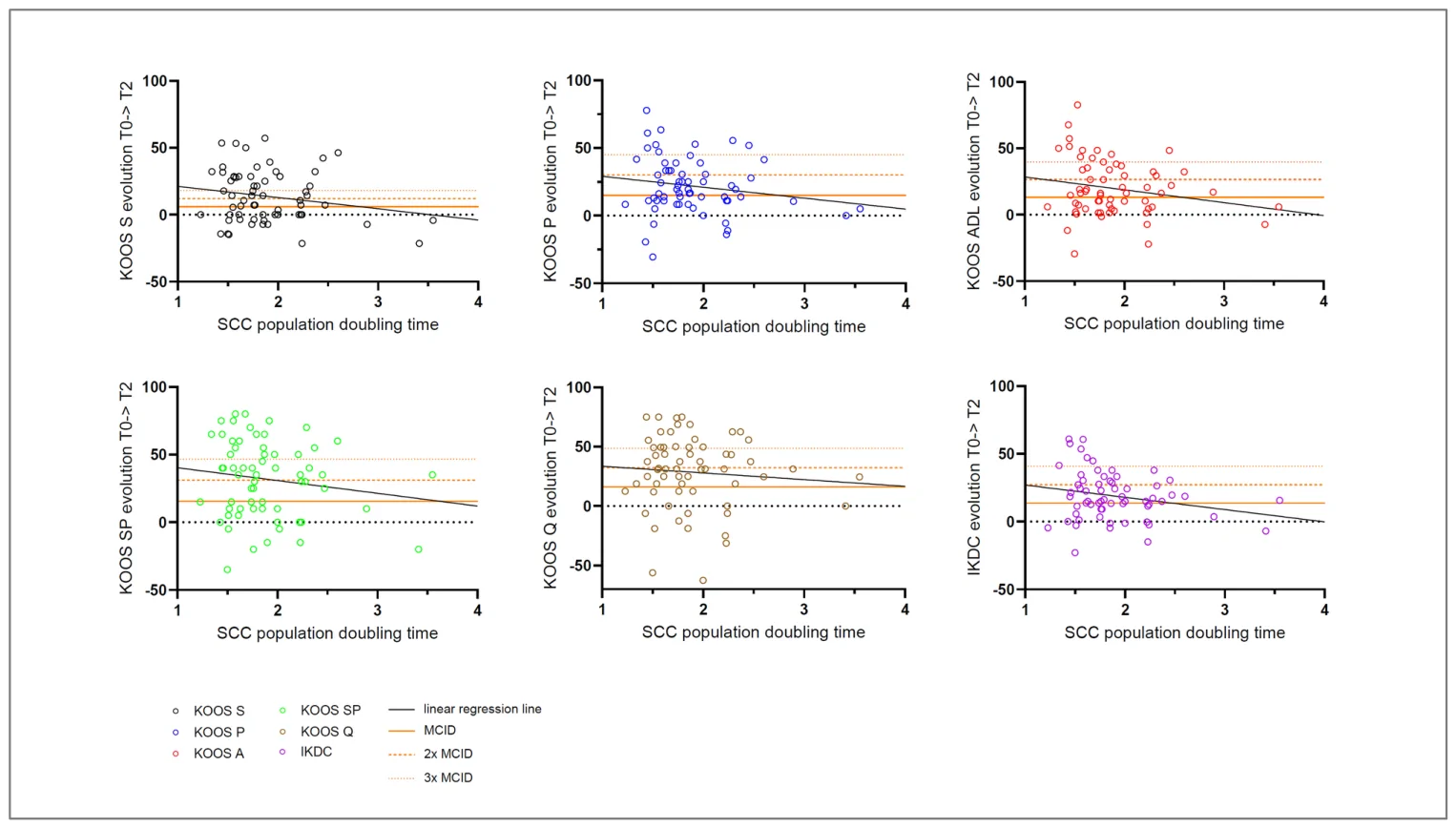
Influence of pre-implantation HAC cell population doubling time (PDT) on achieving clinically meaningful functional improvements. Scatter plots detailing the inverse relationship between the PDT (in days) of the final chondrocyte cell suspension (SCC) and the longitudinal change in clinical scores (ΔPROMs) from baseline (T0) to the final 4-year follow-up (T2). Each panel represents a distinct functional metric (five KOOS subscales and the IKDC score). Individual patient trajectories are plotted alongside simple linear regression lines. The black dotted horizontal line indicates no change from baseline (ΔPROM = 0). Solid and dashed orange lines represent the established Minimal Clinically Important Difference (MCID) and its multiples (2× and 3× MCID), delineating objective thresholds for substantial clinical benefits. KOOS, Knee Injury and Osteoarthritis Outcome Score (S, Symptoms; P, Pain; ADL, Activities of Daily Living; SP, Sport and Recreation Function; Q, Quality of Life); IKDC, International Knee Documentation Committee; MCID, Minimal Clinically Important Difference; SCC, chondrocyte cell suspension.

Further quantitative analyses revealed a significant inverse correlation between the in vitro PDT values and longitudinal improvements in patient-reported outcomes. To clearly visualize this relationship, patient-specific changes in all five KOOS subscales and overall IKDC scores (ΔPROM) from baseline (T0) to the final 4-year follow-up (T2) were plotted directly against their respective PDT values prior to cell therapy implantation (Figure 6).

The stratification of these clinical trajectories using established MCID thresholds, including rigorous 2× and 3× MCID benchmarks, highlights a critical kinetic boundary. Specifically, the data demonstrates that the probability of achieving a substantial, clinically meaningful recovery (defined as an improvement of ≥2× MCID) diminishes markedly when the cellular PDT value exceeds a threshold of 2.1 days (Figure 6). This finding strongly suggests that slower-proliferating chondrocyte populations inherently possess a reduced regenerative potential, translating to a diminished clinical benefit.

This kinetic limitation parallels the previously observed cellular biomass constraints, wherein patients yielding an ICY of fewer than 10^5^ chondrocytes exhibited a similarly depressed probability of achieving optimal functional recovery (Figure 4 and Figure 5). Collectively, these distinct threshold effects strongly implicate both PDT and ICY as highly relevant biological potency proxies. These objective GMP cell manufacturing metrics can thus effectively serve as prognostic indicators, stratifying patients based on their intrinsic biological likelihood of achieving a superior clinical trajectory following second-generation ACI.

### 3.7. Impact of Cryopreservation Media Switch on HAC Cell Population Doubling Times

Given the clinical significance of the 2.1-day kinetic threshold, subsequent analyses were conducted to isolate the specific GMP manufacturing variables directly influencing PDT values. Utilizing an expanded dataset encompassing raw GMP manufacturing metrics from 95 patient samples, the impact of differing cryopreservation protocols on post-thaw HAC proliferative capacity was rigorously evaluated.

Firstly, the proliferative capacity of the cells was tracked by calculating the PDT values both prior to cryopreservation and after thawing. Significantly (*p* < 0.001) higher PDT values were reported for the final cell therapy product (SCC) than for the cellular active substance (HAC), suggesting that cryopreservation is associated with a reduction in subsequent cellular proliferative capacity (Figure S2).

**Figure 7:**
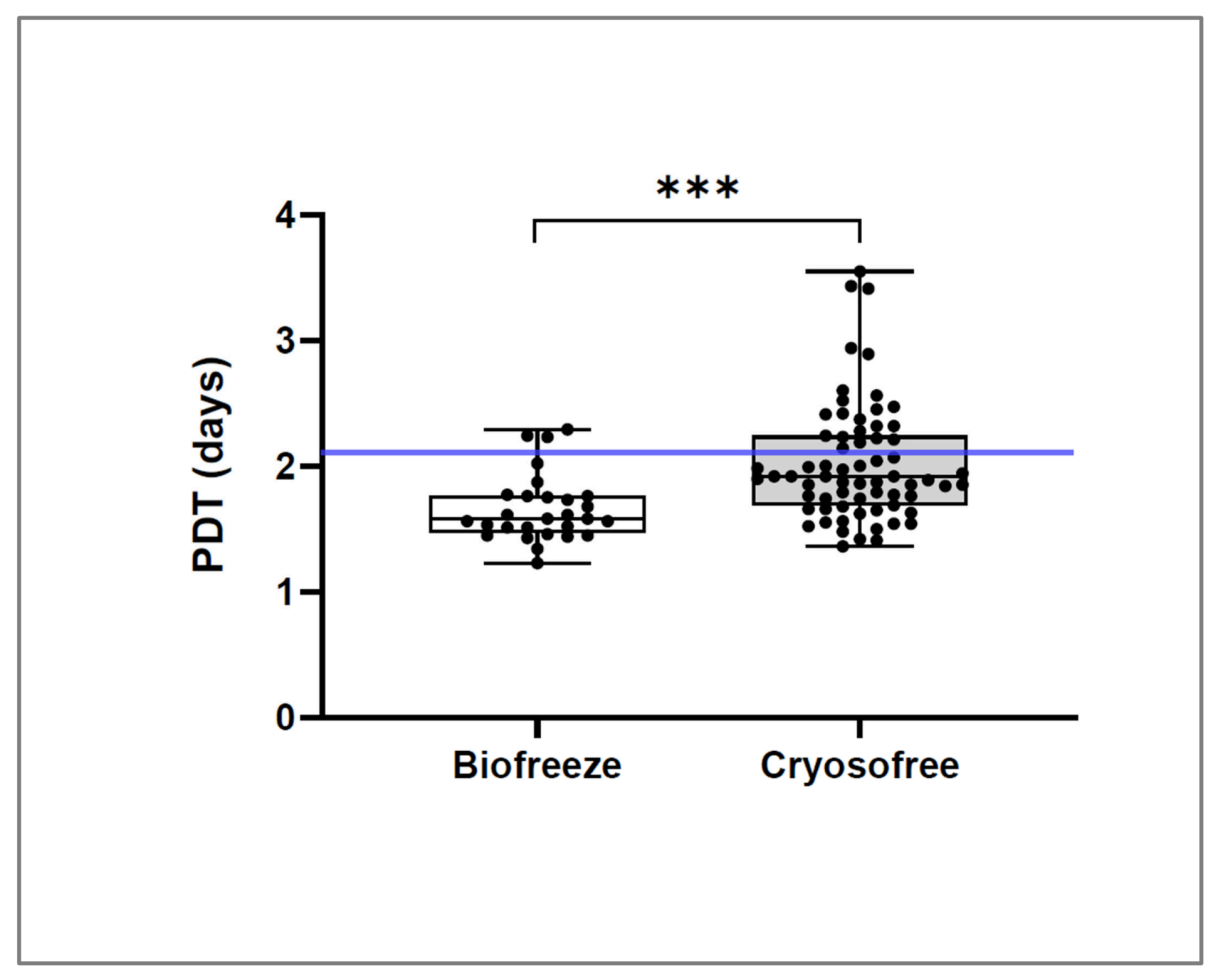
Impact of the different cryopreservation media on post-thaw GMP cellular expansion kinetics. Box-and-whisker plots comparing the population doubling time (PDT, in days) of human articular chondrocytes expanded following cryopreservation in either Biofreeze (n = 28) or in CryoSOfree (n = 67). Individual patient samples are overlaid as black dots. Within the boxes, the central horizontal line represents the median and the outer boundaries denote the interquartile range (IQR), while the whiskers encompass the full data range (minimum to maximum). The solid blue horizontal line represents the critical 2.1-day PDT threshold identified for optimal clinical efficacy. *** *p* < 0.001.

Notwithstanding, the mid-study forced transition between cryopreservation agents further demonstrated a profound and statistically significant effect on subsequent cellular kinetics (*p* < 0.001). As illustrated in Figure 7, chondrocytes expanded following cryopreservation with the original medium, Biofreeze^®^ (n = 28), exhibited a lower median PDT and a more restricted interquartile range.

Within the Biofreeze group of the studied cohort, only 10% of patient samples exceeded the critical 2.1-day threshold (Figure 7). Critically, implementation of the alternative cryopreservation agent, CryoSOfree™ (n = 67), yielded a significantly higher median PDT accompanied by substantially greater inter-sample variability (Figure 7). Consequently, the proportion of patient HAC cultures surpassing the suboptimal > 2.1-day PDT threshold increased markedly to 35% in the CryoSOfree group (Figure 7). These findings highlight that procedural alterations during the GMP cryopreservation phase can substantially influence post-thaw expansion kinetics, significantly increasing the probability of yielding a biologically compromised cellular product that falls outside the optimal therapeutic window.

Finally, the clinical impact of the reported cryopreservation reagent transition was further evaluated by comparing the proportion of patients achieving ≥ 2× MCID in the Biofreeze versus CryoSOfree groups. Generally, a univariate linear regression analysis evaluating the impact of the cryopreservation media transition did not find significant predictive relationships between the specific cryoprotectant agent utilized during GMP bioprocessing (Biofreeze versus CryoSOfree) and the subsequent clinical and radiological trajectories of the patient cohort (Table S2). Notwithstanding, it was shown that in the Biofreeze group (where 90% of samples remained below the 2.1-day PDT threshold, Figure 7), 78% of patients achieved a 2× MCID in KOOS Pain at T2. Conversely, in the CryoSOfree group (where 35% exceeded the 2.1-day PDT threshold, Figure 7), this success rate dropped to 54% (*p* = 0.038). Thus, and most importantly, these data confirm that the endured switch in ancillary manufacturing reagents was associated with a higher frequency of attenuated clinical trajectories.

## 4. Discussion

The successful translation of autologous cell therapies, such as ACI, necessitates a comprehensive evaluation of the entire therapeutic continuum, from the biological quality of the initial cartilage tissue harvest to the patient’s long-term clinical trajectory [4,25]. Although the ex vivo GMP manufacturing protocol in this cohort was rigorously standardized, unavoidable exogenous variables, such as supply chain-mandated alterations in proprietary reagents (cryopreservation agent, culture medium supplements), introduced kinetic fluctuations during HAC expansion. Consequently, the observed heterogeneity in clinical outcomes highlights how extrinsic modifications during the cell therapy bioproduction process can significantly impact, and ultimately reflect, the intrinsic biological regenerative potential of individual patient chondrocytes.

Our findings empirically demonstrate that the biologic potency of the implanted cells, specifically defined by the initial absolute cell yield from the cartilage biopsy and the cellular proliferative capacity quantified by the PDT, exerts a direct influence on patient-reported outcomes following second-generation ACI. The data indicate that suboptimal ICY (specifically <10^5^ cells/biopsy) and attenuated in vitro proliferation rates are strongly associated with inferior trajectories in KOOS Pain and Activities of Daily Living (ADL) scores across the postoperative intervals. Of note, low ICY values may be attributable to small biopsy size, cartilage quality or cell density in the harvested tissue, the in vitro tissue enzymatic digestion process, or underlying clinical conditions. Despite the high level of surgical attention for biopsy size standardization, further improving ICY values most probably requires stringent optimization and standardization of the entire pre-expansion phase, both from surgical and GMP manufacturing viewpoints.

Crucially, the identification of a distinct kinetic threshold, wherein a PDT value exceeding 2.1 days is associated with a significantly diminished probability of achieving clinically meaningful functional improvement, suggests that this metric may be considered as a promising prognostic biomarker. However, due to a limited cohort size, this value requires further confirmation on larger and more diverse patient populations. Collectively, these findings underscore the critical vulnerability of both the initial cartilage biopsy and the subsequent in vitro HAC expansion phase. They emphasize that optimizing and safeguarding the proliferative vigor of the cellular payload is paramount for the ultimate clinical success and structural durability of the ACI procedure.

### 4.1. Delineating the Transversal Nature of Clinical Cell Therapy

Unlike conventional biopharmaceuticals, living cellular products are inherently dynamic, making their therapeutic potency highly susceptible to the microenvironmental stresses imposed during ex vivo isolation, expansion, and cryopreservation [8,11,14,21]. Because the transition of chondrocytes from their native ECM to a static, two-dimensional in vitro environment inevitably introduces the risk of phenotypic drift and metabolic strain [3,8,16], our findings highlight the necessity for a seamless “vein-to-vein” integration [26,27]. Optimal functional restoration is fundamentally predicated on a meticulously optimized bioengineering pipeline that actively mitigates these ex vivo biological stresses [28].

Historically, product release criteria for cell therapies have leaned heavily on fundamental safety and identity metrics [4,12]. By validating specific in vitro kinetic parameters, most notably the PDT, as direct prognostic biomarkers, this study provides the empirical framework necessary to evolve current GMP manufacturing standards [28]. Specifically, this data supports the implementation of dynamic, biologically relevant potency assays as formal release criteria for ACI cell therapy manufacturing, ensuring that only metabolically vigorous and highly competent HAC cellular payloads are cleared for surgical implantation [11,29,30,31].

### 4.2. Imperatives of Multidisciplinary and Interdisciplinary Collaboration

The successful clinical translation of a complex Advanced Therapy Medicinal Product (ATMP), such as ACI cell therapies, necessitates multidisciplinary collaboration to comprehensively optimize the continuum of patient care [4,32,33]. Transitioning to GMP-compliant bioprocesses requires a deep mutual understanding of critical process parameters (CPPs) and critical quality attributes (CQAs), ensuring that any procedural modifications, such as alterations in cryopreservation media, undergo exhaustive, controlled, and validated comparability studies so the intrinsic regenerative quality of the HAC cellular payload is not compromised [34,35].

Concurrently, the post-operative clinical ecosystem plays a vital role in capturing high-fidelity, longitudinal data on patient-reported functional trajectories and objective structural imaging outcomes [36,37]. This iterative flow of information enables the statistical identification of biological prognostic indicators, such as the predictive ICY or kinetic PDT thresholds, and facilitates the continuous refinement of GMP manufacturing protocols required to translate complex cellular therapeutics into standard, highly reliable clinical practice [10].

### 4.3. Study Robustness Through Clinical and Manufacturing Consistency

Despite the relatively constrained sample size of the studied patient cohort, a fundamental methodological strength of this prospective study resides in its stringent clinical and GMP biomanufacturing homogeneity. In the field of orthopedic regenerative medicine, inter-operator variability frequently acts as a substantial source of statistical noise [7]. However, for all enrolled subjects in this study, the entire surgical continuum, encompassing the precise anatomical location and depth of the initial cartilage tissue harvest, the meticulous debridement of the chondral defect, and the final surgical delivery of the ACI cell therapy, was executed by a single senior orthopedic surgeon (R.M.) [4].

Parallel to this clinical uniformity, the entirety of the HAC cellular processing, ex vivo expansion, and rigorous quality control (QC, sub-contracted) testing was conducted by mandate under the responsibility of a singular, centralized, in-house GMP manufacturing facility [4]. This tightly controlled, unidirectional pipeline, spanning from the surgical theatre directly to the cell culture cleanrooms, effectively mitigates confounding variables such as discrepancies in transport logistics, enzymatic digestion times, and surgical handling that frequently dilute the statistical power and obscure the findings of multicenter or multi-operator clinical trials [4,25,38].

Consequently, this high degree of procedural and GMP manufacturing standardization allows us to attribute the residual heterogeneity observed in long-term clinical outcomes with far greater confidence to the intrinsic biological variance and regenerative potential of the patient-specific chondrocytes [27].

Nevertheless, this study also starkly illustrates that even within a highly controlled institutional framework, extrinsic bioprocessing factors must be subjected to continuous, rigorous scrutiny to prevent the introduction of uncharacterized variability [38]. Specifically, our data demonstrate that seemingly innocuous modifications to standard operating procedures (SOPs), such as a manufacturer-mandated transition between commercially available cryopreservation media, can act as a disruptive factor in the kinetic profile of the final cellular therapy product (Figure 7). The significant alteration in post-thaw PDT associated with this reagent change highlights the extreme physiological sensitivity of living biotherapeutics to their ex vivo microenvironment.

Ultimately, the overarching methodological rigor maintained throughout this cohort provides a robust foundation for our conclusions. By systematically neutralizing exogenous technical variables, this study maximizes the signal-to-noise ratio, thereby significantly enhancing the internal validity and translational relevance of the correlations observed between strictly defined in vitro kinetic parameters and longitudinal, patient-reported functional trajectories.

### 4.4. Pertinence of Retained Evaluation Parameters and Endpoints

The analytical parameters and clinical endpoints selected for this study embody a comprehensive, translational “vein-to-vein” paradigm [27,28,39]. Within the manufacturing phase, the selected in vitro metrics function as essential CPPs and surrogate potency assays to assess the therapeutic product’s regenerative competence [11,30,32,40].

ICY serves as a paramount upstream metric; a depressed yield threatens expansion logistics and suggests compromised cellular quality [3,4,16,17,25]. This constrained yield often reflects the inherent phenotypic status of the low-cell density donor tissue [18]. Specifically, theoretically “healthy” cells harvested from a joint already burdened by a focal chondral defect may implicitly suffer from a localized pathological ‘field effect’ that blunts baseline quality [8,17,29].

Concurrently, the PDT provides a dynamic measure of proliferative kinetics, reflecting the cell population’s vitality and senescence profile [4,21,35]. A prolonged PDT may indicate a pathophysiological shift toward cellular senescence [3], often accompanied by a Senescence-Associated Secretory Phenotype (SASP) involving pro-inflammatory cytokine and matrix metalloproteinase (MMP) secretion [17,20]. Implanting such a payload risks lacking metabolic vigor and could actively catalyze graft degradation by perpetuating catabolic inflammation [8,14,15].

Furthermore, quantifying chondrogenic activity via *ACAN* and *COL2A1* expression validates cellular identity and functional fidelity [12,40]. This requires 3D cell pellet cultures to purposefully drive redifferentiation, simulating in vivo matrix deposition potential [3,7]. Finally, monitoring endpoint confluence serves as a practical logistical control to ensure cells are harvested during the optimal logarithmic growth phase prior to surgical delivery.

Translating to the clinical arena, validated patient-reported outcome measures (PROMs) like the KOOS and IKDC are firmly anchored in evidence-based orthopedics [9]. They directly quantify pain mitigation and functional recovery, serving as patient-centric arbiters of intervention success [41]. Complementing these, the objective MOCART score provides an indispensable high-resolution morphological assessment of structural graft integration and subchondral integrity [6,7], establishing a robust, bimodal evaluation methodology that precisely balances patient perception with concrete structural evidence [41,42].

### 4.5. The Need for a Rational Approach to GMP Quality Control

The clinical data presented herein advocate for a highly nuanced, evidence-based paradigm shift regarding quality control (QC) and product release in GMP ACI cell therapy manufacturing, challenging the utility of applying universally rigid specifications across all biological metrics [10,13]. The robust predictive correlations observed between the ICY, PDT, and long-term clinical outcomes firmly establish these kinetic factors as paramount CQAs. Conversely, the absence of a discernible clinical correlation for late-stage *ACAN*/*COL2A1* transcriptional expression and endpoint culture confluence necessitates a strategic recalibration of how these specific parameters are weighted in the release process [4,29,30,43].

Imposing overly dogmatic release thresholds on biological parameters that lack a validated link to in vivo efficacy introduces a severe risk of rejecting viable, potentially curative cellular preparations [12,35,44]. In the context of autologous cell therapies, an unnecessary batch rejection carries profound ethical and logistical weight; it dramatically inflates costs and denies the patient a timely therapeutic intervention [10,11,45]. A more rational, risk-based manufacturing approach dictates prioritizing the kinetic metrics that demonstrably predict patient functional recovery [34]. Consequently, non-predictive metrics should be reclassified as “for information only” (FIO) or as in-process monitoring points [12,29,46]. This stratified QC strategy fosters a robust pipeline that maximizes operational efficiency without compromising biological safety [13,47].

To actualize this shift, manufacturers could formalize PDT and ICY as primary CQAs directly considered before batch release, while petitioning regulatory bodies to relegate static biosynthetic markers to FIO status [19]. Moreover, the stark variation in PDT values resulting from the transition to the CryoSOfree cryopreservation medium underscores the necessity for regulators to mandate kinetic comparability studies whenever a manufacturer amends their approved supply chain [4,19].

This clinically validated emphasis on PDT values underscores the acute vulnerability of living biotherapeutics to seemingly minor raw material variations [14]. Any introduction of novel ancillary materials must be rigorously evaluated for their specific impact on cellular kinetics [11]. Recognizing PDT as a foundational CQA necessitates a highly resilient contingency infrastructure [13], ensuring that unexpected supply chain constraints do not force clinically detrimental process deviations [46]. Manufacturers must thus preemptively validate secondary reagents to ensure uninterrupted clinical manufacturing [48,49,50].

The significant kinetic divergence observed following the reported cryopreservation media transition illustrates the limitations of standard viability testing during process alterations. From a regulatory perspective, such changes dictate rigorous comparability exercises [48]. Our clinical data strongly suggest that traditional post-thaw viability is an insufficient surrogate for cellular potency during these assessments [48]. Therefore, dynamic kinetic metrics, specifically the use of PDT, must be mandated as primary endpoints in comparability protocols [13].

Finally, while the identified PDT threshold of 2.1 days serves as a robust prognostic indicator, it should not be viewed as an absolute binary for batch rejection. Rather, it identifies a high-risk population for whom the probability of a superior clinical outcome is significantly reduced, supporting the transition toward a ‘risk-based’ release strategy.

### 4.6. Comparison with Other Cell Therapy Approaches and Clinical Trials

The direct translational link established here between in vitro biological kinetics and long-term clinical efficacy can be contextualized alongside other leading cell-based orthopedic paradigms [12,51,52], despite inherent differences in patient populations and manufacturing methods limiting direct comparisons. An initial comparison is the “nose-to-knee” approach utilizing autologous nasal septum chondrocytes [40,53]. As demonstrated in pivotal trials, this distinct cell source possesses an inherently superior, plasticity-resistant proliferative capacity [53]. Furthermore, these protocols typically utilize fresh, non-cryopreserved cultures, thereby mitigating freeze–thaw-related senescence and facilitating an optimized PDT. This kinetic superiority robustly aligns with our central thesis and reinforces the concept that mitigating extrinsic bioprocessing stressors is paramount for overarching efficacy [53].

Furthermore, our results hold translational pertinence against advanced commercial cartilage bio-products [3,54,55]. Technologies such as Spherox, which have been validated through rigorous Phase II/III clinical trials [3,30], intentionally utilize a 3D architecture to preserve the functional chondrogenic phenotype of the cells prior to implantation [8,54]. The consistent longitudinal data from these trials demonstrate that a highly standardized, bioengineered cellular product yields predictable in vivo regeneration [54]. Similarly, robust 10-year prospective evaluations of third-generation matrix-induced autologous chondrocyte implantation (MACI) corroborate that a biologically vigorous cellular payload drives successful structural repair and sustained clinical outcomes [3,55].

Ultimately, while specific cellular sources and in vitro expansion methodologies diverge across proprietary protocols, the collective body of clinical evidence converges on a singular principle: the intrinsic biological quality and kinetic potency of the final cellular product is a primary determinant of long-term clinical success and graft durability, alongside surgical, biomechanical, and patient-specific factors [3,9,52,53,54,55]. The reproducible success across these varied regenerative approaches underscores the necessity for the field to pivot its focus toward the dynamic biologic characteristics of the cells themselves [4,53,54,55].

### 4.7. Contextualizing the Dissociation of Clinical and MRI Outcomes

Our analysis revealed a clear absence of statistically significant correlations between any of the isolated in vitro biological determinants and long-term magnetic resonance imaging (MRI) outcomes, as quantified by the MOCART score. This divergence underscores a well-documented clinical-radiological paradox: the frequent dissociation between macroscopic morphological tissue repair and actual symptomatic or functional recovery [6,12,36,41,56]. Specifically, these findings do not bring forth appropriate evidence linking optimal cellular metrics with improved macroscopic repair tissue quality.

Standard morphological MRI sequences and the associated MOCART scoring system evaluate gross structural indices of the implanted graft, such as defect fill volume and marginal integration [8,42]. However, these conventional imaging modalities lack the resolution to interrogate the ultrastructural composition, biochemical makeup, or the true biomechanical competence of the regenerated ECM [7,39,57]. Consequently, it is highly plausible that the resultant neocartilage, while appearing morphologically intact on standard MRI, may persist functionally as a mechanically inferior fibrocartilage rather than true hyaline cartilage [3,5,10,17,51]. Such tissue lacks the requisite viscoelastic properties to adequately dissipate physiological loads [5,7,8]. Under repetitive mechanical stress, this inadequacy translates to altered load transmission to the subchondral bone, triggering persistent nociceptive signaling and patient-reported functional limitations [5,6,58].

In contrast, validated PROMs (KOOS and IKDC) capture the complex interplay of pain mitigation and joint kinematics, frequently proving to be more sensitive and holistic indicators of therapeutic efficacy than static structural imaging alone [9,37,41]. Ultimately, evaluating ACI success necessitates a multimodal approach that triangulates objective structural assessments with dynamic, patient-centric functional evaluations.

The lack of correlation between MOCART scores and biological parameters highlights the limitations of a purely morphological assessment. Future research should thus prioritize compositional MRI sequences, such as T2 mapping or delayed gadolinium-enhanced MRI of cartilage (dGEMRIC), which provide quantitative data on glycosaminoglycan content and collagen fiber orientation [7]. Such modalities may provide the necessary resolution to visualize ultrastructural differences between grafts produced from high-vigor versus low-vigor chondrocyte populations.

### 4.8. The Role of Chondrogenic Markers and Confluence

The observed absence of a significant prognostic correlation between terminal *ACAN* and *COL2A1* transcriptional expression and longitudinal clinical outcomes represents a notable finding [59]. While the upregulation of these core matrix genes is undeniably essential for hyaline cartilage neogenesis, our data suggest that their isolated in vitro expression levels are not the definitive arbiters of in vivo functional recovery [12,21]. Assessing gene expression via 3D cell pellet culture effectively validates a cell’s baseline chondrogenic plasticity [3]. However, this highly controlled in vitro assay fails to accurately recapitulate the cell’s ultimate biosynthetic trajectory when subjected to the dynamic physiological environment of the synovial joint [17,29,60]. The multi-axial mechanical forces and complex biochemical crosstalk present in vivo orchestrate a mechanotransduction cascade that is far more complex than what can be modeled in a static in vitro culture [3,8,10,17]. Therefore, while a static 3D cell pellet assay successfully confirms foundational epigenetic plasticity, it cannot predict whether the implanted chondrocytes possess the functional resilience required to maintain a hyaline phenotype when subjected to the mechanical forces of the native human knee [5,7,12,51].

Similarly, while achieving a defined percentage of endpoint confluence remains a standard manufacturing quality control parameter to prevent contact inhibition, it demonstrated negligible predictive bearing on the ultimate clinical resolution [43,61]. This strongly suggests that the dynamic kinetic vigor of the HAC cellular payload, quantified by the PDT, is a fundamentally more crucial biological determinant than the static spatial density achieved at the termination of monolayer expansion [26]. A culture that reaches high confluence may do so sluggishly, potentially masking underlying cellular senescence or metabolic exhaustion [14,20]. Conversely, a rapidly dividing population inherently possesses the robust metabolic plasticity and sustained proliferative momentum necessary to survive the surgical transfer, efficiently populate the cartilage defect site, and initiate durable ECM synthesis [15]. Therefore, the absolute yield of metabolically vigorous, highly proliferative cells supersedes simple endpoint culture density as a driver of successful outcomes.

### 4.9. Clinical and Manufacturing Implications

The empirical correlations established herein hold profound translational implications for both the clinical application and GMP bioprocessing of ACI cellular therapeutics [4,14]. Identifying ICY and PDT values as kinetic prognostic biomarkers advocates for their integration into preoperative patient stratification and as biologically relevant release criteria [10,61].

Clinically, these predictive metrics empower orthopedic surgeons to engage in evidence-based shared decision-making regarding inherently variable patient and lesion characteristics [16]. A patient with a critically low ICY or a prolonged PDT could be prospectively counseled on the elevated risk of attenuated recovery, allowing for proactive expectation management and consideration of alternative strategies before implantation [6,29]. Establishing a ‘suboptimal’ cellular profile (e.g., ICY < 10^5^ cells or PDT > 2.1 days) could trigger a multidisciplinary review to discuss potential pivot strategies (e.g., new biopsy harvest) prior to arthrotomy [6]. This clear contingency algorithm avoids subjecting patients to a second-stage surgery biologically predisposed to yield attenuated functional recovery [25].

Concurrently, from a biomanufacturing perspective, these kinetic parameters provide actionable data to refine cell culture and cryopreservation protocols [13,18,26]. By monitoring PDT as a dynamic CPP, GMP manufacturing facilities can proactively intervene to ensure a high-potency final product [12,15,30]. Adopting these biologically validated thresholds shifts the field toward a personalized, predictive, and biologically customized strategy for cartilage regeneration [19,27,28,37].

Furthermore, the predictive power of ICY and PDT was found to be more pronounced at T2 (~4 years) than at T1 (2 years). This suggests that the initial cellular payload’s biological quality dictates the long-term durability and fatigue resistance of the repair tissue, whereas short-term outcomes may be more influenced by surgical factors and early rehabilitation compliance [6,7,37].

Integrating these thresholds also carries substantial health economic implications [3,13,32,44]. Second-generation ACI is a resource-intensive intervention [9,10]. Implanting a product with a PDT > 2.1 days reduces the probability of achieving a 2× MCID in pain reduction, potentially exposing the healthcare system to compounding costs of persistent morbidity and revision arthroplasty [9]. Conversely, rejecting HAC batches based on non-predictive morphological markers wastes the biopsy investment; thus, adopting validated kinetic criteria ensures that high-cost therapies are allocated to patients with the requisite biological machinery for durable joint restoration [9,19,45,46].

Within a GMP Quality Management System (QMS), the rigid binary of ‘pass/fail’ release criteria is often inadequate for complex autologous cell therapies [27,40]. We advocate translating the 2.1-day PDT threshold into a formalized Out-of-Trend (OOT) action limit rather than an immediate Out-of-Specification (OOS) rejection. Reaching this limit should trigger an investigation and clinical notification, allowing teams to collaboratively assess the risk-benefit ratio of the HAC batch formulation versus culture termination, thereby embedding data-driven risk management into batch disposition [33,34,35].

### 4.10. Limitations and Future Research Perspectives

Despite robust methodological controls, this prospective study presents inherent limitations. Foremost, the constrained cohort size and single-institution design may limit the epidemiological generalizability of our findings [9]. Given the biological heterogeneity in patient-specific cellular kinetics, future validation within a larger, multicenter cohort is essential to definitively corroborate these thresholds. Furthermore, the PDT may be influenced by patient age, biopsy quality, and in vitro culture conditions, complicating the disentanglement of biological from process-related effects.

Additionally, this analysis did not exhaustively capture all covariates known to influence articular cartilage repair [6]. Patient-specific variables, such as baseline comorbidities, joint biomechanics, activity levels, and rehabilitation adherence, act as potential unmeasured confounders that influence clinical trajectories and regression model variance [6,10,14]. Furthermore, intraoperative variables, including defect topography and concomitant pathologies, play a substantial role in cell therapy product integration [5,6,18]. Specifically, our results were not adjusted for factors like patient age, lesion size and location, defect type, or previous surgeries. Future investigations must integrate these multifaceted covariates to construct comprehensive, multi-dimensional predictive models [10].

Structural evaluations were restricted to conventional morphological MRI sequences using the MOCART score. The absence of compositional MRI modalities (e.g., T2 mapping or dGEMRIC) prevents definitive conclusions regarding the ultrastructural and biochemical quality of the regenerated ECM relative to cellular kinetic thresholds. Additionally, while the identified kinetic thresholds (ICY > 10^5^ cells, PDT < 2.1 days) serve as robust prognostic indicators, the observed inter-patient variability necessitates interpreting these parameters as enhanced probabilities for clinical success rather than absolute guarantees. Future predictive models must account for this variability when establishing hard release criteria.

Finally, static monolayer expansion and standard analytical assays cannot fully recapitulate the dynamic mechanobiological microenvironment of the native joint [12]. Future translational research must incorporate advanced in vitro modeling, such as dynamic bioreactor systems, to better elucidate how baseline cellular quality translates into functional tissue repair [3,8]. Moreover, expanding the analytical panel to include comprehensive multi-omic profiling and immunophenotyping could unveil novel prognostic biomarkers and strategies to salvage sub-optimal cellular payloads [10,11,15]. Exploring specific manufacturing variants (e.g., hypoxic culture, FGF-2 supplementation) may enhance reliable CQA fulfillment [17,20,21,34,61]. Continued investigation into next-generation bio-functionalized scaffolds and advanced cell delivery systems is also warranted to optimize spatial retention, drive in situ cell differentiation, and streamline surgical implantation [3,5,8,10].

## 5. Conclusions

This prospective longitudinal study effectively bridges the translational divide within regenerative orthopedics by establishing a direct, empirical correlation between GMP-controlled cell metrics and the long-term clinical trajectories of patients following second-generation ACI. By leveraging a highly standardized institutional framework, characterized by a single-surgeon continuum and a centralized GMP biomanufacturing facility, the research successfully neutralizes exogenous technical variables, allowing the intrinsic biological potential of patient-specific chondrocytes to be clearly visualized.

The primary significance of this work lies in the promising identification of ICY and PDT as kinetic prognostic biomarkers of ACI patient positive clinical evolution. Specifically, the data demonstrate that an ICY value exceeding 10^5^ cells and a PDT value of less than 2.1 days are associated with markedly superior improvements in pain relief, joint function, and activities of daily living over a follow-up period averaging over four years. Crucially, the findings reveal a notable dissociation between conventional in vitro benchmarks and actual in vivo success, as neither terminal chondrogenic marker expression (*ACAN*, *COL2A1*) nor endpoint HAC culture confluence showed significant correlations with long-term patient-reported or structural outcomes. This lack of correlation underscores the need for a strategic recalibration of QC protocols, advocating for a risk-based approach that prioritizes dynamic kinetic vigor over static biosynthetic or spatial assessments. Overall, our results show that kinetic metrics of the cells (PDT and ICY) outperform classical chondrogenic phenotype markers in predicting the clinical response after ACI.

Overall, the study highlights the extreme physiological sensitivity of living biotherapeutics to GMP manufacturing alterations. Recognizing therapy-specific biological thresholds should empower clinicians and manufacturers to move away from empirical “one-size-fits-all” treatments toward a highly personalized and predictive strategy for joint preservation, ensuring that only the most metabolically competent cellular payloads are utilized to drive durable cartilage regeneration.

## Figures and Tables

**Table 1:** Baseline demographic, clinical, and surgical characteristics of the patient cohort. Continuous variables (age, body mass index, and defect size) are presented as mean ± standard deviation (SD). Categorical variables (sex, lesion location, lesion type, and surgical technique) are presented as absolute frequencies with percentages (%). DFO, distal femoral osteotomy; HTO, high tibial osteotomy.

Variable	Value/Frequency
Number of patients	67
Sex, n (%)	Male: 38 (57%)/Female: 29 (43%)
Age at implantation, years (mean ± SD)	25.1 ± 8.3
Body mass index, kg/m^2^ (mean ± SD)	23.3 ± 3.6
Defect size after debridement, cm^2^ (mean ± SD)	5.4 ± 2.5
Lesion location, n (%)	Femoral condyles: 37 (55%)/Patellofemoral joint: 30 (45%)
Lesion type, n (%)	Chondral: 25 (37%)/Osteochondral: 42 (63%)
Two-layer “sandwich” technique, n (%)	42 (63%) (DFO: 6 (9%)/HTO: 35 (52%))

## Data Availability

The datasets generated and supporting the conclusions of this article are available within the manuscript and its associated Supplementary Files. Further inquiries can be directed to the corresponding authors.

## Supplementary Materials

The following supporting information can be downloaded at https://www.mdpi.com/article/10.3390/life16081297/s1. Figure S1. MRI-based MOCART scores at the final follow-up; Figure S2. Comparison of population doubling time values between HAC and SCC chondrocyte populations; Table S1. Multiple regression analysis of predictors of clinical outcomes; Table S2. Univariate linear regression analysis of the association between cryoprotectant type and clinical outcomes; Table S3. Details of the rehabilitation program.

## Abbreviations

The following abbreviations are used in this manuscript:

ACI	Autologous Chondrocyte Implantation
ADL	Activities of Daily Living
ACAN	Aggrecan
ATMP	Advanced Therapy Medicinal Product
BMI	Body Mass Index
COL2A1	Collagen Type II Alpha 1 Chain
CPP	Critical Process Parameter
CQA	Critical Quality Attribute
DFO	Distal Femoral Osteotomy
dGEMRIC	Delayed Gadolinium-Enhanced MRI of Cartilage
ECM	Extracellular Matrix
FIO	For Information Only
GMP	Good Manufacturing Practices
HAC	Human Articular Chondrocytes
hPL	Human Platelet Lysate
HTO	High Tibial Osteotomy
ICY	Initial Chondrocyte Yield
IPC	In-Process Control
IKDC	International Knee Documentation Committee
IQR	Interquartile Range
KOOS	Knee Injury and Osteoarthritis Outcome Score
MACI	Matrix-Induced Autologous Chondrocyte Implantation
MCB	Master Cell Bank
MCID	Minimal Important Clinical Difference
MMP	Matrix Metalloproteinase
MOCART	Magnetic Resonance Observation of Cartilage Repair Tissue
OOS	Out-of-Specification
OOT	Out-of-Trend
PDT	Population Doubling Time
PROM	Patient-Reported Outcome Measure
QC	Quality Control
QMS	Quality Management System
QOL	Quality of Life
RT-qPCR	Real-Time Quantitative Polymerase Chain Reaction
SASP	Senescence-Associated Secretory Phenotype
SCC	Chondrocyte Cell Suspension
SOP	Standard Operating Procedure
T1	Follow-up at 2 years
T2	Final follow-up at 4.2 ± 1.7 years
