# Supplementary material for: Predicting ACI Outcomes with GMP In-Process Control Kinetic Metrics: Initial Chondrocyte Yield and Population Doubling Time as Prognostic Clinical Biomarkers
Source: Life (Basel). 2026 Aug 6;16(8):1297. doi: 10.3390/life16081297 (PMC13514264; doi:10.3390/life16081297)
Supplement: Supplementary file 1 [file life-16-01297-s001.zip › life-4460495-supplementary.pdf]

Supplementary Data: Predicting ACI Outcomes with GMP In-Process Control Kinetic Metrics: Initial Chondrocyte Yield and Population Doubling Time as Prognostic Clinical Biomarkers

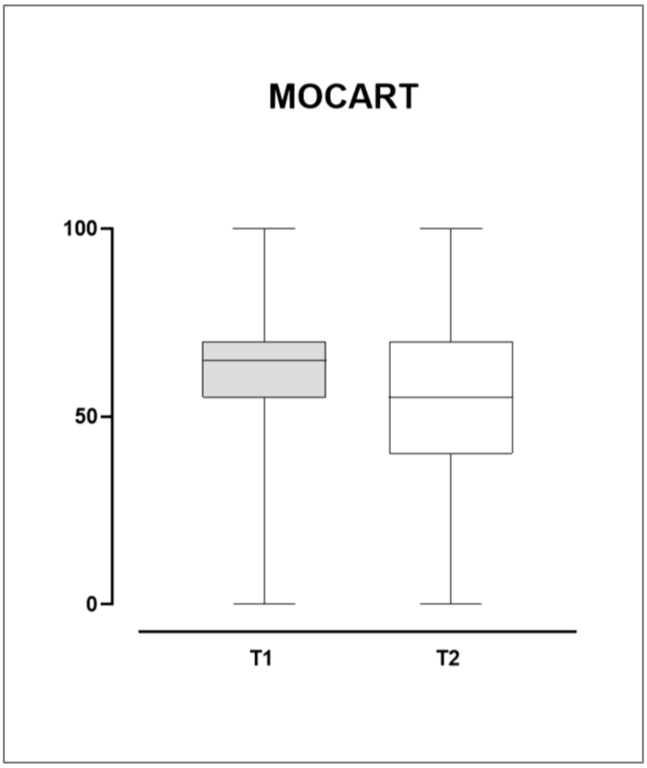

Figure S1. Longitudinal structural evaluation via MRI-based MOCART scores. Objective, high-resolution morphological assessment of the regenerated tissue following second-generation autologous chondrocyte implantation (ACI; n = 45). The box-and-whisker plot delineates the Magnetic Resonance Observation of Cartilage Repair Tissue (MOCART) scores recorded at the final follow-up interval (T2, 4.2 ± 1.7 years). This validated scoring system facilitates the evaluation of critical structural indices, including graft integration with native cartilage, defect fill volume, and subchondral bone integrity. Within the plot, the central horizontal line represents the median value, the box indicates the interquartile range (IQR), and the whiskers represent the overall range of the data distribution.

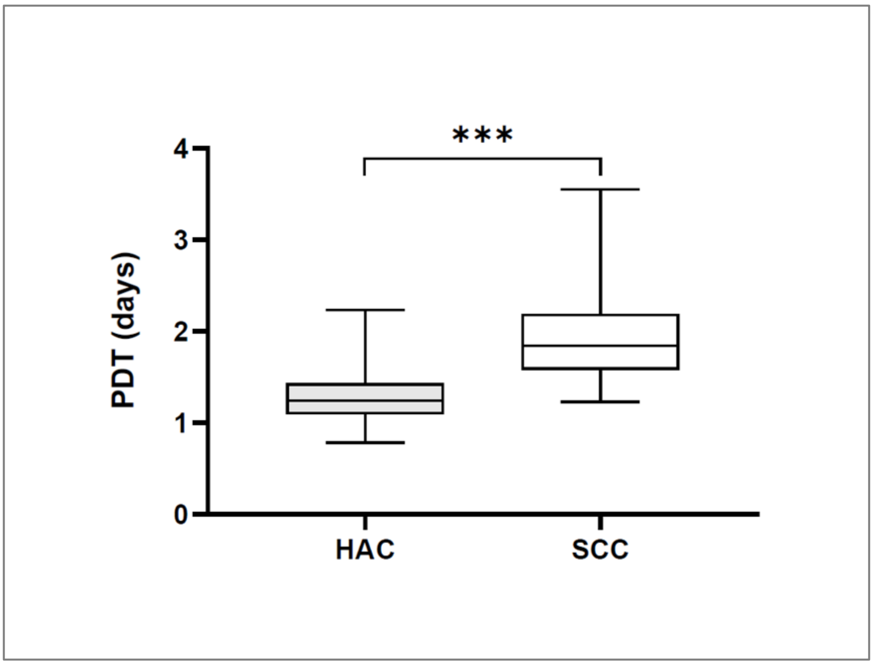

Figure S2. Comparative analysis of proliferative kinetics between primary HAC and post-thaw SCC populations (n = 66). Differential population doubling time (PDT) values observed in human articular chondrocytes (HAC) during the primary expansion phase (cellular active substance) compared to the final chondrocyte cell suspension (SCC) following cryopreservation and recovery. The box-and-whisker plots delineate the growth kinetics of these cell populations, with central horizontal lines representing median values, box extremities indicating the interquartile range (IQR), and whiskers capturing the full data distribution. Primary HAC populations demonstrated a significantly lower median PDT, reflecting superior proliferative vigor prior to the exogenous metabolic and osmotic stressors imposed by the freeze-thaw cycle. A statistically significant increase in PDT was observed in the SCC final product (*** $p < 0.001$), accompanied by markedly greater inter-sample variability. These findings highlight the profound impact of cryopreservation on cellular kinetic momentum and emphasize the importance of monitoring proliferative capacity as a dynamic indicator of metabolic vigor during the GMP biomanufacturing process.

Data analysis note: The differences in sample size between Figures 6, 7, and S2 are due to the different inclusion criteria applied for each analysis. In detail, Figure 7 (n = 73) represents the patients with a minimum follow-up of 2 years (≥ 2 years). After exclusion of 6 patients according to exclusion criteria after implantation (study flowchart), 67 patients remained for the final clinical analysis. Figure 6 (n = 95) includes all implanted patients with available biological manufacturing data, including patients with less than 2 years of clinical follow-up. This analysis was part of a broader evaluation of the GMP manufacturing process and included available cryopreservation-related data, independently of the availability of data on long-term clinical outcomes. Figure S2 (n = 66) includes the post-thaw SCC samples with available PDT measurements. One patient was excluded from this analysis because the PDT value was considered an outlier, most likely related to a technical issue rather than a biological characteristic.

Table S1. Multivariate linear regression analysis [1] correlating in vitro GMP biomanufacturing parameters with longitudinal clinical and structural outcomes (between T0–T1 and T0–T2). The table delineates the predictive associations between primary biological Critical Quality Attributes (CQAs), encompassing initial cell yield (ICY), chondrogenic marker expression, terminal culture confluence, and population doubling time (PDT), and validated clinical and radiological endpoints.

Outcome Type	Value Type	Low *COL2A1* Expression		Low *ACAN* Expression		Low Cell Confluence Percentage		Insufficient Isolated Cell Yield		Increased Population Doubling Time	
		T0–T1	T0–T2	T0–T1	T0–T2	T0–T1	T0–T2	T0–T1	T0–T2	T0–T1	T0–T2
**KOOS Symptoms**<br>n = 53<br>$R^2 = 0.11$	β coefficient<br>*p*-value	0.003<br>*ns*	0.005<br>*ns*	0.081<br>*ns*	–0.005<br>*ns*	–0.3<br>*ns*	–0.25<br>*ns*	11.78<br>*ns*	63.56<br>**	–18.18<br>*ns*	–17.07<br>*ns*
**KOOS Pain**<br>n = 53<br>$R^2 = 0.26$	β coefficient<br>*p*-value	0.003<br>*ns*	0.001<br>*ns*	0.021<br>*ns*	0.035<br>*ns*	–0.26<br>*ns*	–3.34<br>*ns*	–67.5<br>*ns*	88.83<br>***	–16.13<br>*ns*	–23.09<br>*
**KOOS ADL**<br>n = 53<br>$R^2 = 0.32$	β coefficient<br>*p*-value	0.003<br>*ns*	0.015<br>*ns*	–0.026<br>*ns*	–0.008<br>*ns*	–0.195<br>*ns*	–0.23<br>*ns*	–1.22<br>*ns*	107.84<br>***	–14.84<br>*ns*	–21.71<br>*
**KOOS Sport and Recreation**<br>n = 53<br>$R^2 = 0.15$	β coefficient<br>*p*-value	0.038<br>*ns*	0.042<br>*ns*	–0.017<br>*ns*	–0.097<br>*ns*	–0.11<br>*ns*	–0.28<br>*ns*	4.12<br>*ns*	92.30<br>*	–12.99<br>*ns*	–22.75<br>*ns*
**KOOS QOL**<br>n = 53<br>$R^2 = 0.03$	β coefficient<br>*p*-value	–0.013<br>*ns*	–0.002<br>*ns*	0.176<br>*ns*	0.076<br>*ns*	–0.33<br>*ns*	–0.39<br>*ns*	–14.29<br>*ns*	48.72<br>*ns*	–16.01<br>*ns*	–27.79<br>*ns*
**IKDC**<br>n = 54<br>$R^2 = 0.13$	β coefficient<br>*p*-value	0.015<br>*ns*	0.019<br>*ns*	0.022<br>*ns*	0.017<br>*ns*	–0.11<br>*ns*	–0.31<br>*ns*	4.99<br>*ns*	14.40<br>*ns*	–13.46<br>*ns*	–26.12<br>**
**MOCART**<br>n = 45<br>$R^2 = 0.11$	β coefficient<br>*p*-value	0.009<br>*ns*	0.01<br>*ns*	0.16<br>*ns*	0.17<br>*ns*	0.42<br>*ns*	0.11<br>*ns*	–4.68<br>*ns*	16.27<br>*ns*	19.53<br>*ns*	15.95<br>*ns*

[1] Standardized coefficients (β) and associated *p*-values are reported for each multivariate model to characterize the magnitude and statistical significance of these relationships across all KOOS subscales, the IKDC score, and MOCART structural assessments. Thresholds defining low expression or altered cellular parameters were: *COL2A1* fold change < 50, *ACAN* fold change < 10, isolated cell yield < 1×10^5 cells per cartilage biopsy, and PDT > 2.1 days. The coefficient of determination (R^2) indicates the proportion of total variance explained by the regression models, while n denotes the specific number of patient datasets (n = 53) utilized for the analysis. ADL, activities of daily living; QOL, quality of life; IKDC, international knee documentation committee; KOOS, knee injury and osteoarthritis outcome score; PDT, population doubling time; MOCART, magnetic resonance observation of cartilage repair tissue; ns, non-significant ($p > 0.05$). * $p < 0.05$, ** $p < 0.01$, *** $p < 0.001$.

Data analysis note: Figure 1 provides an overall representation of the study cohort and summarizes the global results. Patients were not excluded from this overview in case they had missing data for any of the individual follow-up parameters. For example, if KOOS data were missing at T1 for patient A, this patient was still included in the analyses involving the available data at T0 and T2. In contrast, Table S1 presents the results of the multiple linear regression analyses. For these analyses, only patients with complete datasets for all variables were considered in each specific model. Therefore, patients with missing values for any of the variables required for a regression model were excluded from that particular analysis.

Table S2. Simple linear regression analysis [1] evaluating the impact of the cryopreservation media transition on longitudinal clinical outcomes, reported as the association between PDT and clinical outcomes (β coefficients and *p*-values) according to the cryoprotectant. The table quantifies the predictive relationship between the specific cryoprotectant agent utilized during Good Manufacturing Practice (GMP) bioprocessing (Biofreeze® versus CryoSOfree™) and the subsequent clinical outcomes of the patient cohort.

**Biofreeze®**				**CryoSOfree®**			
**Outcome Type**	**Value Type**	**T0–T1**	**T0–T2**	**Outcome Type**	**Value Type**	**T0–T1**	**T0–T2**
**KOOS Symptoms**<br>n = 28	β coefficient<br>R^2<br>*p*-value	−7.10<br>0.01<br>*ns*	−15.68<br>0.05<br>*ns*	**KOOS Symptoms**<br>n = 35	β coefficient<br>R^2<br>*p*-value	−6.59<br>0.02<br>*ns*	−2.93<br>0.005<br>*ns*
**KOOS Pain**<br>n = 28	β coefficient<br>R^2<br>*p*-value	−0.84<br>0.0002<br>*ns*	−16.59<br>0.03<br>*ns*	**KOOS Pain**<br>n = 35	β coefficient<br>R^2<br>*p*-value	−6.28<br>0.02<br>*ns*	−4.20<br>0.01<br>*ns*
**KOOS ADL**<br>n = 28	β coefficient<br>R^2<br>*p*-value	−13.49<br>0.04<br>*ns*	−33.52<br>0.12<br>*ns*	**KOOS ADL**<br>n = 35	β coefficient<br>R^2<br>*p*-value	−3.86<br>0.006<br>*ns*	−2.18<br>0.004<br>*ns*
**KOOS Sport and Recreation**<br>n = 28	β coefficient<br>R^2<br>*p*-value	−1.52<br>0.0002<br>*ns*	−23.25<br>0.04<br>*ns*	**KOOS Sport and Recreation**<br>n = 35	β coefficient<br>R^2<br>*p*-value	−11.22<br>0.04<br>*ns*	−3.32<br>0.004<br>*ns*
**KOOS QOL**<br>n = 28	β coefficient<br>R^2<br>*p*-value	13.21<br>0.02<br>*ns*	−16.25<br>0.02<br>*ns*	**KOOS QOL**<br>n = 35	β coefficient<br>R^2<br>*p*-value	1.04<br>0.0003<br>*ns*	1.90<br>0.0008<br>*ns*
**IKDC**<br>n = 28	β coefficient<br>R^2<br>*p*-value	−1.46<br>0.0004<br>*ns*	−16.75<br>0.05<br>*ns*	**IKDC**<br>n = 35	β coefficient<br>R^2<br>*p*-value	−7.17<br>0.04<br>*ns*	−4.16<br>0.02<br>*ns*

[1] Standardized coefficients (β) and *p*-values are provided for each outcome measure, characterizing the direct influence of extrinsic reagent modifications on therapeutic efficacy. The coefficient of determination (R^2) reflects the proportion of outcome variance attributable to the choice of cryopreservation medium, while n represents the total number of patient datasets included in the analysis. These results underscore the critical physiological sensitivity of living biotherapeutics to ancillary material variations and supply chain-mandated alterations within the GMP manufacturing pipeline. ADL, activities of daily living; QOL, quality of life; IKDC, international knee documentation committee; KOOS, knee injury and osteoarthritis outcome score; PDT, population doubling time; ns, non-significant ($p > 0.05$).

Table S3. Details of the rehabilitation program followed by the study participants following implantation of the cellular product [1].

Phase	Objectives	Restrictions	Range of Motion	Exercises/Activities
Preoperative	–	–	–	–
**Phase 1** 0–7 days post-surgery	• Avoid loading > 20% body weight (BW). • Avoid shear forces across the articular surface. • Maintain lower limb joint mobility, muscle tone, and circulation. • Reduce postoperative pain and swelling.	Weight bearing: ≤ 20% BW on the operated knee, using crutches.	Passive and active: 0°–30°	• Knee: CPM (0°–30°): Initiated 12–24 h after surgery for at least 1 h/day. Active knee flexion and extension (0°–30°). • Isometric contractions of the quadriceps, hamstrings, adductors, and gluteal muscles. • Ankle: Active dorsiflexion and plantar flexion.
**Phase 2** 2–3 weeks post-surgery	• Achieve 90° knee flexion and full painless extension. • Avoid loading > 30% BW. • Restore active quadriceps control. • Reduce postoperative pain and swelling.	Increase weight bearing from ≤ 20% BW (weeks 1–2) to 30% BW (week 3), with crutches.	Increase from 30° (week 2) to 90° (week 3), passive and active.	• Continue Phase 1 exercises. • Knee: Patellar mobilization in different directions. Progressive active flexion and extension to achieve 0°–90° by week 3. CPM during physiotherapy visits if necessary. • Isometric quadriceps, hamstrings, adductors, and gluteal exercises. • Straight leg raises (SLR) in supine position. • Ankle: Active dorsiflexion and plantar flexion. • Hip: Active flexion, abduction, adduction, and extension.
**Phase 3** 4–6 weeks post-surgery	• Achieve 125° knee flexion and maintain full painless extension. • Avoid loading > 60% BW.	Increase weight bearing from 40% BW (week 4) to 60% BW (week 6), with crutches.	Increase from 90° (week 4) to 125° (week 6), passive and active.	• Continue Phase 1 and 2 exercises. • SLR in supine, with external hip rotation and side-lying. • Stretching: Hamstrings and calf muscles. • Ankle exercises against resistance. • Hip strengthening against resistance. • Core strengthening. • Introduce recumbent cycling (week 5). • Introduce hydrotherapy: Deep-water walking, shallow-water walking, heel raises, squats, cycling, scissor movements, and proprioception.
**Phase 4** 7–12 weeks post-surgery	• Achieve full ROM. • Begin pain-free walking without crutches. • Begin pain-free cycling. • Perform home and supervised exercises.	Increase weight bearing from 80% BW (week 7) to full weight bearing (week 8). Discontinue crutches thereafter.	Full active ROM (weeks 7–8).	• Continue Phase 2 and 3 exercises. • Knee: Begin squats at 8 weeks. • Weight-bearing proprioception. • Introduce upright cycling (weeks 9–12). • Continue more advanced hydrotherapy (weeks 11–12).

**Phase 5**<br>3–6 months post-surgery	• Normal pain-free gait without crutches or brace.<br>• Ability to climb and descend stairs without pain.<br>• Perform all strengthening and proprioceptive exercises at full weight bearing.	Full weight bearing without crutches.	Full and pain-free.	• Continue Phase 3 and 4 exercises.<br>• Increase cycling duration and intensity.<br>• Introduce elliptical trainer and rowing machine.

[1] It applies to patients undergoing autologous chondrocyte implantation (ACI) for isolated chondral lesions. For osteochondral lesions treated using the Sandwich technique (autologous cancellous bone grafting combined with ACI), the rehabilitation protocol was identical except for weight-bearing restrictions. In these patients, weight bearing was limited to 5 kg on the operated limb for the first 8 weeks postoperatively to protect osseous graft incorporation, after which progression followed the standard protocol.
